# Ground and Excited States of 3d and 4d Transition Metals: Computational Insight into Atomic Properties

**DOI:** 10.1021/acs.jpca.5c05650

**Published:** 2025-09-22

**Authors:** Ebtisam M. Z. Telb, Nuno M. S. Almeida, Bradley K. Welch, Angela K. Wilson

**Affiliations:** Department of Chemistry, 3078 Michigan State University, East Lansing, Michigan 48864, United States

## Abstract

Effective strategies for the computational prediction and ordering of the ground and low-lying excited states of 3d and 4d transition metal atoms can be difficult to achieve due to their high density of states, multiple spins, and narrow energy gaps. In this work, the energy manifolds for first- and second-row transition metal atoms were investigated using the super-correlation consistent Composite Approach (s-ccCA) and several multireference wave function approaches in combination with the correlation consistent basis sets. Scalar relativistic effects were also incorporated. The impact of these choices on the prediction of these energies was analyzed. Multireference configuration interaction with the Davidson correction (MRCI+Q) offers great reliability and accuracy for most transition metal atoms when extrapolated to the complete basis set limit. In a comparison of methodologies, s-ccCA results in the lowest deviation from experiment.

## Graphical abstract

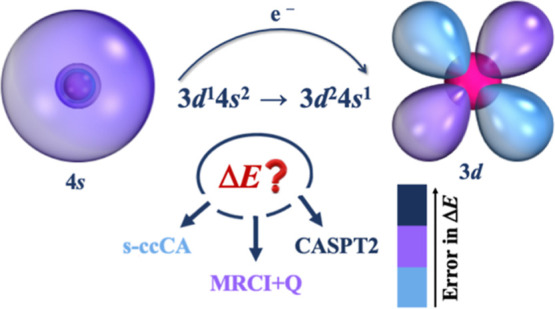

## 1 Introduction

Transition metal compounds have wide-ranging industrial and technological applications, encompassing catalytic conversion,^1–3^ optical devices,^4^ magnets,^5,6^ photocatalysis, and photovoltaics^7,8^ due to their unique properties. To gain insight into the electronic structure and bonding of transition metals, an accurate characterization of atomic energy levels and ground and excited states can be useful. Accurately describing the ground and excited states of transition metals is crucial for catalysis, as many catalytic routes require excited-state species to proceed to the optimal reaction pathway.

While there are numerous prior studies employing ab initio methods in which the spectroscopic features of transition metal ground and excited states of atoms have been predicted,^9–35^ a comprehensive investigation with a systematic approach that can describe the ground and several excited states of all of the 3d and 4d transition metals has not been performed. This is not surprising, as describing the multireference (MR) nature for many of the excited states can be quite complicated due to their complex electronic structure and energetic manifolds. However, single reference methods have been widely used to investigate the low-lying d^*x*^s^2^ → d^*x*+1^s^1^ excitation energies of 3d and 4d transition metal atoms.^10–15,17,19–22^,^31,36–39^ To provide a number of examples, in 1989, Raghavachari and Trucks calculated the excitation energies of Sc–Cu transition metal atoms using fourth-order Møller–Plesset perturbation theory (MP4) and quadratic configuration interaction (QCISD­(T)).^15^ QCISD­(T) consistently performed better for all excitation energies, showing a mean deviation from the experimental value of 3.2 kcal mol^–1^ after including relativistic corrections. Density functional theory calculations using BLYP were performed for excitation energies of the first transition series, rendering a larger error of approximately 11.5 kcal mol^–1^ as compared to QCISD­(T).^10^ In other studies, coupled cluster methods showed low errors for the excitation energies of the 3d and 4d transition metal atoms.^11,13,14,17,31^ Balabanov and Peterson reported the most accurate d^*x*^s^2^ → d^*x*+1^s^1^ excitation energies of 3d transition metal atoms using CCSD­(T), CCSDT, and CCSDTQ extrapolated to the complete basis set (CBS) limit, resulting in a maximum error of −0.61 kcal mol^–1^ for a^3^F excited state of Ni.^14^ Peterson et al. computed the d^*x*^s^2^ → d^*x*+1^s^1^ excitation energies of 4d transition metal atoms using CCSD­(T)/CBS, showing that the error in the calculated excitation energies for early 4d transition metal atoms can be significant, reaching 4 kcal mol^–1^ for a^5^D excited state of the Mo atom.^17^ Much of this error can be attributed to the need for even higher levels of correlation treatment than those provided by CCSD­(T). The error in the a^5^D excited state of the Mo atom was reduced to −1.1 kcal mol^–1^ by incorporating both 3d correlation and higher-levels of electron correlation.^31^

The MR nature of many 3d and 4d transition metal-containing molecules is well-known, arising from their partially filled d orbitals and leading to strong electron correlation and multiple low-lying electronic states that require an approach that can describe static correlation.^40–45^ Thus, a number of prior studies were carried out to investigate the low-lying states of 3d and 4d transition metal atoms using MR wave function approaches (refs 9, 13, 16, 18, 24–26, 33–35, 46 ). In prior studies, the excited states of Ni were shown to be particularly challenging to describe due to strong electron correlation and the near degeneracy of a^3^D and a^3^F states for which the *J*-averaged experimental separation is only 0.65 kcal mol^–1^ (refs 9, 13, 14, 16, 18, 19, 26, 32, 46–48 ). Bauschlicher et al. conducted MR configuration interaction singles and doubles (MRCISD) calculations on the a^3^D, a^3^F, and a^1^S states using an atomic natural orbital basis set, demonstrating the impact of the inclusion of 4d orbitals in the active space.^18^ Their calculations rendered errors of −5.3 and 2.3 kcal mol^–1^ for a^3^D → a^3^F and a^3^D→ a^1^S splitting, respectively. Sauri et al. calculated the first six excited states of Ni using the complete active space second-order perturbation theory (CASPT2)/ANO-RCC and two different active spaces: (i) 3d, 4s; (ii) 3d, 4s, 4d orbitals, also correlating the 3s and 3p electrons.^9^ State average calculations were performed for triplet states (15) and singlet states (19). The CASPT2 excitation energies deviated significantly from experimental data in the absence of a second d-shell in the active space, revealing the key role of a doubled d-shell in stabilizing the excitation energies, as previously demonstrated by Andersson and Roos.^16^ For all the states except a^1^S, the error ranged from −6.9 to −11.5 kcal mol^–1^, whereas a substantial error of up to −62.26 kcal mol^–1^ was observed for a^1^S state. However, incorporation of the second d-shell in the active space reduced the error to approximately 4.6 kcal mol^–1^ for the states. The authors calculated the excitation to the a^1^S state, which deviated from the experimental energy by 1.26 kcal mol^–1^, however, it can be reduced further by including the 4p orbital in the active space. The averaged coupled pair functional (ACPF) method was utilized to compute the d^*x*^s^2^ → d^*x*+1^s^1^ excitation energy of 3d transition metal atoms, exhibiting good agreement with the experiment for early transition metals; however, a large error was observed for the late transition metals.^14^ An error of −2.10 kcal mol^–1^ was found for a^3^D → a^3^F excitation energy of the Ni atom.

Raab and Roos calculated the excitation energy of 3d and 4d transition metal atoms using CCSD­(T)/ANO-RCC and CASPT2/ANO-RCC, showing that these two methods generally yielded comparable results with the exception of first-row atoms with less than five 3d electrons.^13^ The outer core electron correlation and active space of the 4s, 4p, 3d, and 4d orbitals were considered in the ACPF and CASPT2 calculations. On the other hand, Alizadeh Sanati and Andrae calculated the first excited states of Sc–Cu, Ag, and Au atoms with MRCI+Q using three different sets of active space: (i) 3s, 3p, 4s, and 3d, (ii) 4s and 3d, and (iii) 4s, 4p, and 3d, demonstrating an overall mean absolute deviation (MAD) error in the range of 1.4 to 3.2 kcal mol^–1^.^26^ Further, Lodi et al. calculated a^4^F and a^2^F excitation energies of Sc to be 2.3 and 2.0 kcal mol^–1^ greater than the experimental energies using MRCI+Q/cc-pwCVQZ-DK and 3d4s4p orbitals in the active space.^25^

Another avenue to calculate accurate energetics for transition metal complexes is via composite schemes. Ab initio composite methods have also been used to describe the spectroscopic and thermodynamic properties of transition metals, enabling a reduced computational cost relative to traditional ab initio methods.^49–52^ One of the most effective composite methods for transition metal species is the correlation consistent Composite Approach (ccCA).^53–55^ The ccCA scheme has been applied extensively to the 3d transition metals,^54^ 4d transition metals,^55,56^ as well as to lanthanide and actinide species.^57–59^ In earlier work on the 3d transition metals, the term “transition metal chemical accuracy” was coined to recognize the average experimental uncertainty of 3 kcal mol^–1^ for a broad range of the best (at the time) enthalpies of formation available for 3d species. For a set of 4d species using a pseudopotential-based ccCA, this overall level of accuracy was obtained as well.

Recently, super-ccCA (s-ccCA)^60–62^ was introduced to better describe the dissociation energies of 3d and 4d molecules in light of new, unprecedented experimental measurements by Merriles and Morse.^63–67^ Compared to its predecessor, ccCA, s-ccCA includes higher-level coupled cluster corrections (full triple, quadruple, and quintuple excitations), which have had a demonstrated impact upon dissociation energies, particularly for 3d species, though still a meaningful impact for 4d species.^61^

In this study, a comprehensive characterization of the ground and several excited states of all of the 3d and 4d transition metal atoms is performed. MRCI, CASPT2, and s-ccCA, are utilized, enabling a comparison of the impact of single- and MR-based methods. The current findings offer valuable insights into the spectroscopic characteristics of transition metal-containing molecules.

## 2 Computational Details

The state-averaged complete active space self-consistent field (SA-CASSCF) method was utilized as the reference wave function for MR calculations.^68^ The symmetry components of each state were incorporated into the active space. For the 3d transition metals, the active space at the CASSCF level, from scandium to copper, consisted of 14 orbitals: 3d, 4d, 4s, and 4p orbitals, included at D_2h_ symmetry, with five of a_g_ symmetry (4s, 3/4d_*z* ^2^_, and 3/4d_*x* ^2^–*y* ^2^_), one of b_1u_ (4p_*z*_), one of b_2u_ (4p_*y*_), one of b_3u_ (4p_*x*_), two of b_1g_ (3/4d_*xy*_), two of b_2g_ (3/4d_*xz*_), and two of b_3g_ (3/4d_*yz*_) symmetries. For zinc, since the 3d orbitals are all filled, they were not included at the CASSCF level. However, the 3d orbitals were still considered for post-CASSCF calculations in the core card (as per MOLPRO 2022 implementation).^69–71^ In addition, for the active space of zinc, 5s, 4d, and 5/6p_*x*,*y*,*z*_ orbitals (a_g_, b_1u_, b_2u_, and b_3u_ symmetries) were also included.

A fully relativistic third-order Douglas–Kroll–Hess Hamiltonian (DKH3) was utilized for all MR calculations of the 3d transition metals. Regarding basis sets, the augmented correlated consistent basis sets at the triple-, quadruple-, and quintuple-ζ (aug-cc-pVXZ-DK, where X = T, Q, and 5) were utilized.^72^ In addition, the augmented polarized weighted core–valence correlation consistent basis sets at the triple-, quadruple-, and quintuple-ζ levels (aug-cc-pwCVXZ, where X = T, Q, and 5) were utilized.^72^ All of the energetic predictions were extrapolated to the CBS limit using the following expression by Peterson et al.^73^

1 $$E\left(X\right)=E_{CBS}+A\;\exp\left[-\left(X-1\right)\right]+B\;\exp\left[-{\left(X-1\right)}^{2}\right]$$

In eq 1, X corresponds to the basis set level: triple-(3), quadruple-(4), or quintuple-(5)­ζ. *E*_CBS_ is the energy at the CBS limit and *A* and *B* are coefficients that are determined in the extrapolation.

For post-CASSCF calculations, two different methodologies were considered for the 3d transition metals: the MR configuration interaction singles and doubles (MRCI) and the CASPT2.^74,75^ The MRCI and CASPT2 calculations incorporated the core electron correlation by including single and double excitations of the core electrons in the active space. For core-MRCI (C-MRCI) calculations from scandium to manganese, four additional 3s and 3p_*x*,*y*,*z*_ (a_g_, b_1u_, b_2u_, and b_3u_ symmetries) orbitals were also included. From iron to copper, only the 3s orbital was considered. The Davidson correction was considered for all atoms and calculations.

The 3s and 3p_*x*,*y*,*z*_ electrons were included in core-CASPT2 (C-CASPT2) calculations for the atoms from scandium to copper. As mentioned previously, for zinc, the core orbitals considered at the C-MRCI+Q and C-CASPT2 levels included the 3s, 3p, and 3d electrons. For the C-CASPT2 calculations, the default shift for the ionization potential electron affinity (IPEA) zeroth-order Hamiltonian, 0.25 au, was used.^76,77^ To avoid weakly coupled intruder state’s interference, an imaginary level shift of 0.2 au was employed.^78,79^

For 4d transition metals, the same active space was considered from yttrium to silver: five a_g_ symmetry (5s, 4/5d_*z* ^2^_, and 4/5d_*x* ^2^–*y* ^2^_), one b_1u_ (5p_*z*_), one b_2u_ (5p_*y*_), one b_3u_ (5p_*x*_), two b_1g_ (4/5d_*xy*_), two b_2g_ (4/5d_*xz*_), and two b_3g_ (4/5d_*yz*_) symmetries. For silver at the CASSCF level, a_g_ (6s) was also included in the active space. In addition, for cadmium as for zinc, the 4d orbitals were not included in the active space, though the 6s, 5d, and 6/7p_*x*,*y*,*z*_ orbitals (a_g_, b_1u_, b_2u_, and b_3u_ symmetries) were added to the active space. A nonrelativistic Hamiltonian was considered for the MR calculations. Augmented correlation consistent basis sets were used for all calculations with a pseudopotential of 28 electrons, again at the triple-, quadruple-, and quintuple-ζ levels, extrapolated to the CBS limit using eq 1.^73,80^

For the post-CASSCF calculations on the 4d transition metal atoms, C-MRCI+Q and C-CASPT2 were also employed.^74,75^ For C-MRCI+Q, from yttrium to technetium, the 4s and 4p_*x*,*y*,*z*_ (a_g_, b_1u_, b_2u_, and b_3u_ symmetries) orbitals were considered. From ruthenium to silver, only the a_g_ (4s) orbital was considered. For silver, having the 6s orbital in the active space led to an exceptional number of integrals and determinants. For example, 1,881,456 determinants were obtained for a ^2^S, a^2^D, and b^2^S states at SA-CASSCF/aug-cc-pwCV5Z-PP. Therefore, the 6s was removed from the active space, and the b^2^S (4d^10^6s^1^) excited state was not addressed with C-MRCI+Q calculations.

For C-CASPT2, from yttrium to palladium, the 4s and 4p_*x*,*y*,*z*_ orbitals were included in all of the calculations. For silver, due to the added 6s orbital in the active space, it was not possible to also include the 4s and 4p_*x*,*y*,*z*_ orbitals at C-CASPT2; however, the core-correlation (4s and 4p_*x*,*y*,*z*_) energy was recovered from the core–valence term (Δ*E*_CV_), calculated with CCSD­(T)/CBS. A similar implementation was previously considered by Phung et al. for CASPT2/CCSD­(T) calculations.^81^ As for zinc, the core correlation for cadmium was recovered by including the 4s, 4p_*x*,*y*,z_, and 4d orbitals at C-CASPT2. The same values of level shift and IPEA shift as those for 3d transition metals were utilized.

s-ccCA calculations were also performed to compare with MR predictions. The ground and single reference excited states were calculated. s-ccCA includes terms up to CCSDTQP, which provides an extensive coupled cluster treatment. Within s-ccCA, additional terms that account for the core–valence correlation and spin–orbit effects were included. The energies obtained by s-ccCA are spin-free. The s-ccCA approach was applied for the lowest-energy spin states that can be characterized by a single leading determinant, using the lowest solution at the Hartree–Fock (ROHF) level as the reference for the computations. The full description and implementation of s-ccCA can be found in ref 60.

MOLPRO 2022 was used for the MR calculations.^71^ MOLPRO 2022 ^71^ and MRCC^82^ were used for s-ccCA. Specifically, MRCC was utilized for calculations of higher-order coupled cluster, i.e., CCSDT, CCSDTQ, and CCSDTQP approaches. The calculated excitation energies were compared to the *J*-averaged experimental energies from the NIST Atomic Spectra Database.^83^

## 3 Results and Discussion

The first five excited states of the 3d and 4d transition metals were predicted for most of the elements, with some exceptions, as will be further discussed. The computed excitation energies of all atoms using s-ccCA, CASSCF, C-CASPT2, and C-MRCI+Q, along with the corresponding *J*-averaged experimental energies, are shown in Tables 1–7. The MADs, mean signed deviations (MSDs), and maximum errors (max. errors) of the excited state energies determined using C-MRCI+Q/CBS and C-CASPT2/CBS relative to experimental energies are provided in Figures 1 and 2 for 3d and 4d transition metals, respectively. The computed excitation energies presented herein were extrapolated to the CBS limit using energies determined at the aug-cc-pwCVXZ (X= T, Q, 5) basis set levels. The energies extrapolated to the CBS limit utilizing aug-cc-pVXZ (X= T, Q, 5) level energies are available in Tables S1–S7. Their MADs, MSDs, and max. errors are depicted in Figures S1 and S2 of the Supporting Information. The errors for computed excitation energies at s-ccCA are also provided in Figures 1 and 2 for the 3d and 4d transition metals. The contributions to the s-ccCA excitation energies are included in Tables S10–S13 in the Supporting Information. The MR diagnostics are also included in Tables S14 and S15.

**Figure 1:**
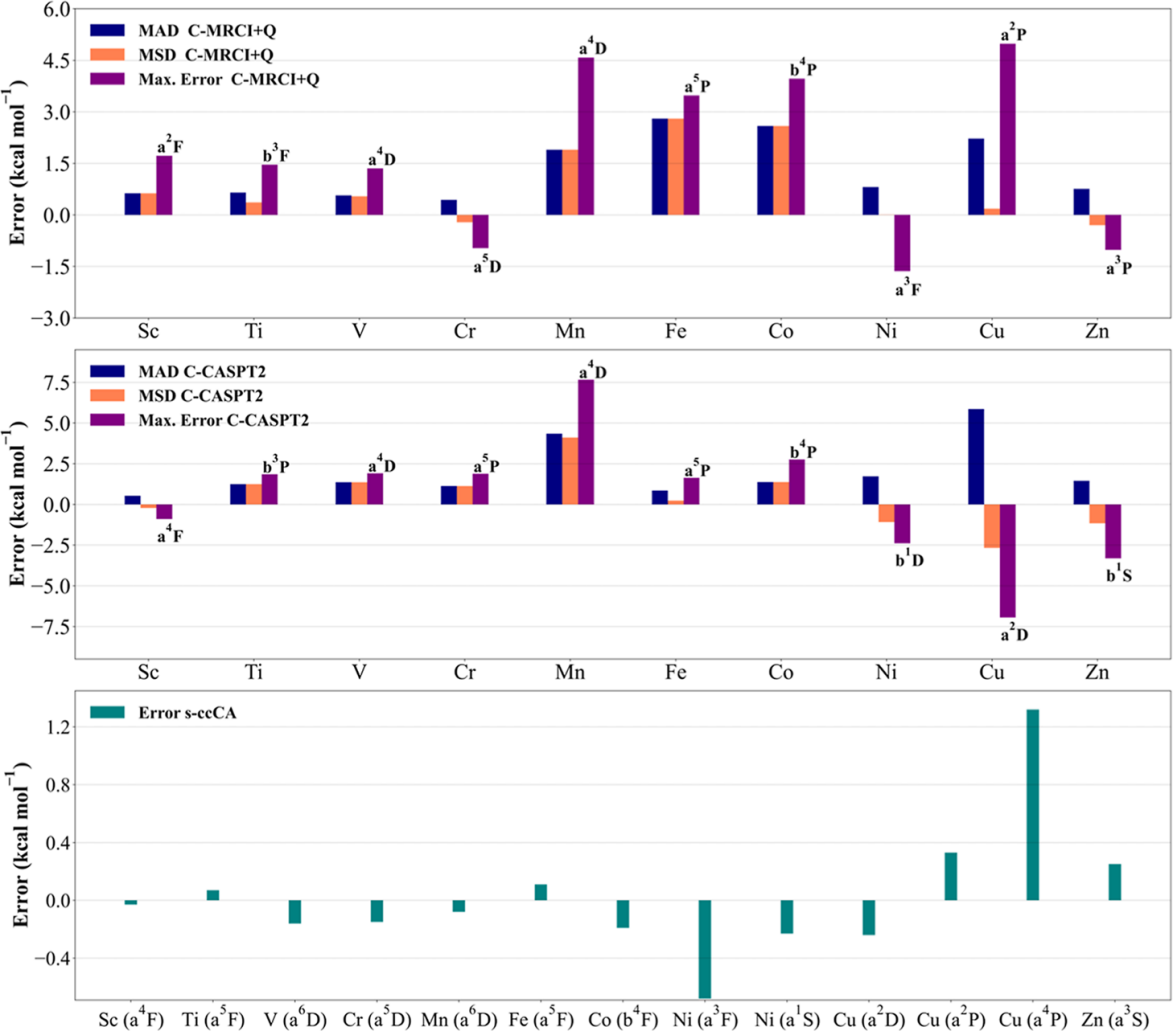
Mean absolute deviation (MAD), mean signed deviation (MSD), and maximum error (max. error) of 3d transition metal excitation energies calculated with C-MRCI+Q/CBS and CASPT2/CBS, and the error in the computed excitation energies using s-ccCA.

**Figure 2:**
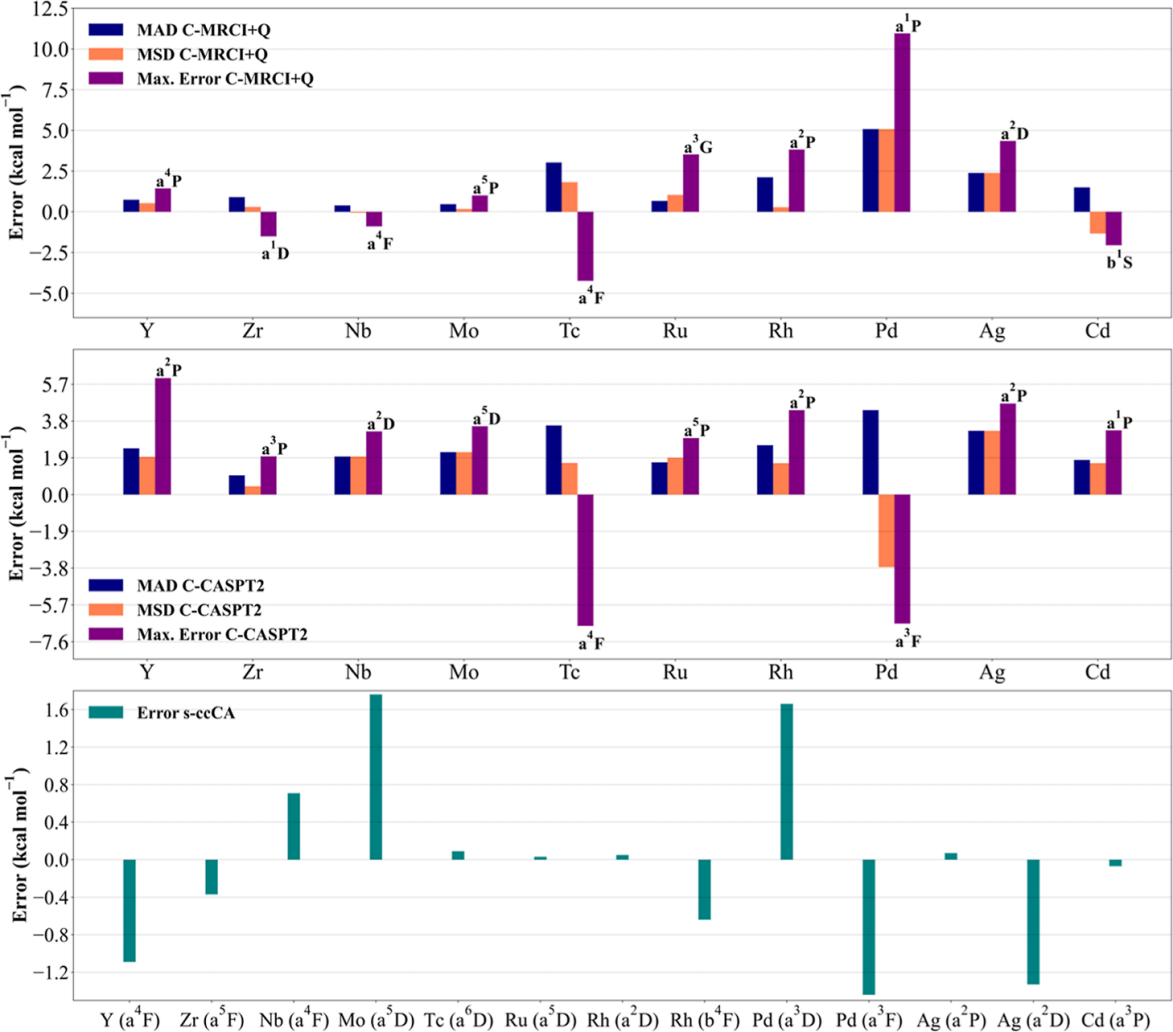
MAD, MSD, and max. error of the 4d transition metal excitation energies calculated at C-MRCI+Q/CBS and CASPT2/CBS, and the error in the computed excitation energies using s-ccCA.

### 3.1 3d Transition Metals

As shown in Tables 1–4, the excitation energies arising from the C-CASPT2 and C-MRCI+Q calculations were significantly improved relative to CASSCF. The latter exhibits substantial deviation from the experimental energies by accounting for mostly static (nondynamic) electron correlation, while C-CASPT2 and C-MRCI+Q have terms characterizing the dynamic correlation. The data in Figure 1 show that C-MRCI+Q/CBS performs well for Sc–Cr, Ni, and Zn elements with a maximum error of 1.72 kcal mol^–1^ obtained for the ^2^F excited state of Sc. However, large max. errors of 4.58, 3.47, 3.96, and 4.98 kcal mol^–1^ were observed for a^4^D, a^5^P, b^4^P, and a^2^P excited states of Mn, Fe, Co, and Cu elements, respectively. The MADs with values of 1.90, 2.80, 2.59, and 2.22 kcal mol^–1^ occurred for C-MRCI+Q/CBS for the Mn, Fe, Co, and Cu elements, respectively. Interestingly, using C-CASPT2/CBS leads to improvement for a^5^P and b^4^P excited states of Fe and Co elements, decreasing the max. error to 1.63 and 2.76 kcal mol^–1^, and the MADs to 0.85 and 1.37 kcal mol^–1^, respectively (see Figure 1). Conversely, Mn and Cu excitation energies at C-CASPT2/CBS deteriorate compared to those at C-MRCI+Q/CBS, rendering a max. error of 7.66 and −6.95 kcal mol^–1^ for a^4^D and a^2^P excited states, respectively. However, the MAD for Mn at the C-MRCI+Q level is less than 2 kcal mol^–1^.

Comparing our findings to those from previous studies, Alizadeh Sanati and Andrae computed the a^4^F and a^2^F excitation energies of Sc using C-MRCI+Q/def2-QZVPP (including the 3s, 3p, 4s, and 3d orbitals in the active space), rendering errors of 2.5 and 3.9 kcal mol^–1^, respectively.^26^ In another study, Raab and Roos^13^ and Balabanov and Peterson^14^ reported lower errors of 0.07 and 0.32 kcal mol^–1^ for the a^4^F excited state of Sc at C-CASPT2/ANO-RCC and ACPF/CBS levels, respectively. ACPF/CBS^14^ excitation energies also agree with experimental data for the first excited states of Ti, V, and Cr atoms, whereas the corresponding C-CASPT2/ANO-RCC^13^ energies vary substantially. For instance, errors of −3.13, −4.04, and 5.47 kcal mol^–1^ from experiment were obtained for a^5^F, a^6^D, and a^5^D excitation energies of Ti, V, and Cr atoms with C-CASPT2/ANO-RCC (see Tables 1 and 2). However, our results show errors of 0.75, 1.55, and 1.14 kcal mol^–1^ with C-CASPT2/CBS and errors of 2.34, 2.96, and −0.40 kcal mol^–1^ with CASPT2/aug-cc-pwCVTZ-DK, respectively. Interestingly, Alizadeh Sanati and Andrae computed the first excited states of Ti, V, and Cr atoms at MRCI+Q/def2-QZVPP, and their results align closely with those obtained herein for the C-MRCI+Q/CBS calculations.^26^ The authors included the 3s, 3p, 4s, and 3d orbitals in the active space for Ti and V, and 4p, 4s, and 3d orbitals in the active space for Cr.

The investigated excited states of Mn included the first seven states to maintain the degeneracy of the ground and excited states, where a^4^D and b^4^D excited states have mixed character. As an example, in the absence of the b^4^D, the five configurations representing the a^4^D state are not degenerate with the SA-CASSCF reference wave function. Adding the b^4^D state to the SA-CASSCF reference wave function led to a degeneracy of a^4^D state configurations and a significant improvement in the calculated a^4^D excitation energy. For example, the error of a^4^D excitation energy decreased from 9.30 to 4.93 kcal mol^–1^ with C-MRCI+Q/aug-cc-pwCV5Z-DK. The b^4^D excitation energy with C-MRCI+Q/CBS was not determined due to the added computational cost; instead, it was determined with C-CASPT2 at the CBS limit, which resulted in an error of −0.81 kcal mol^–1^ (see Table 2).

**Table 1:** Relative Ground and Excitation Energies (in kcal mol^–1^) of Sc, Ti, and V Elements with s-ccCA, CASSCF/CBS, C-CASPT2/CBS, and C-MRCI+Q/CBS Levels^h^

	Sc						Ti						V					
	a^2^D	a^4^F	a^2^F	b^4^F	a^4^D	b^2^D	a^3^F	a^5^F	a^1^D	a^3^P	b^3^F	a^1^G	a^4^F	a^6^D	a^4^D	a^4^P	a^2^G	a^2^P
methods	3d^1^4s^2^	3d^2^4s^1^	3d^2^4s^1^	3d^1^4s^1^4p^1^	3d^1^4s^1^4p^1^	3d^1^4s^1^4p^1^	3d^2^4s^2^	3d^3^4s^1^	3d^2^4s^2^	3d^2^4s^1^	3d^3^4s^1^	3d^2^4s^2^	3d^3^4s^2^	3d^4^4s^1^	3d^4^4s^1^	3d^3^4s^2^	3d^3^4s^2^	3d^3^4s^2^
CASSCF	0	42.22	52.36	40.43	39.59	39.06	0	28.44	25.51	29.19	44.58	39.86	0	16.57	37.63	33.82	36.06	47.32
C-CASPT2	0	32.01	43.37	45.09	45.38	45.09	0	19.37	21.74	25.65	34.00	34.68	0	7.20	25.58	28.43	31.00	39.87
s-ccCA	0	32.88					0	18.65					0	5.49				
C-MRCI+Q	0	33.86	44.30	45.20	46.13	45.70	0	19.66	19.89	23.53	34.20	33.80	0	6.61	25.02	26.79	30.65	38.95
Expt.^a^	0	32.91	42.58	45.11	45.83	45.61	0	18.58	20.11	23.80	32.74	34.01	0	5.65	23.67	26.86	30.56	38.56
C-MRCI+Q-Expt.	0	0.95	1.72	0.09	0.30	0.09	0	1.08	–0.22	–0.27	1.46	–0.21	0	0.96	1.35	–0.07	0.09	0.39
C-CASPT2-Expt.	0	–0.90	0.79	–0.02	–0.45	–0.52	0	0.79	1.63	1.85	1.26	0.67	0	1.55	1.91	1.57	0.44	1.31
previous work^b^	0	0.33^c^	3.9^e^				0	0.34^c^	1.2^e^	0.2^e^			0	0.21^c^				
		0.32^d^	2.0^f^					0.33^d^						0.32^d^				
		2.5^e^						–1.2^e^						–4.04^g^				
		2.3^f^						–3.13^g^										

a J-averaged experimental energies from ref 83.

b Theor—Expt; from ref 83.

c Peterson coupled cluster composite approach from ref 14.

d ACPF values from ref 14.

e MRCI+Q from ref 26.

f CASPT2 from ref 25.

g CASPT2 from ref 13.

h The energies were extrapolated using aug-cc-pwCVXZ-DK (X = T, Q, and 5).

**Table 2:** Relative Ground and Excitation Energies (in kcal mol^–1^) of Cr, Mn, and Fe Elements with s-ccCA, CASSCF/CBS, C-CASPT2/CBS, and C-MRCI+Q/CBS Levels^h^

	Cr					Mn								Fe			
	a^7^S	a^5^S	a^5^D	a^5^G	a^5^P	a^6^S	a^6^D	a^8^P	a^4^D	a^6^P	a^4^G	a^4^P	b^4^D	a^5^D	a^5^F	a^3^F	a^5^P
methods	3d^5^4s^1^	3d^5^4s^1^	3d^4^4s^2^	3d^5^4s^1^	3d^5^4s^1^	3d^5^4s^2^	3d^6^4s^1^	3d^5^4s^1^4p^1^	3d^6^4s^1^	3d^5^4s4p^1^	3d^5^4s^2^	3d^5^4s^2^	3d^5^4s^2^	3d^6^4s^2^	3d^7^4s^1^	3d^7^4s^1^	3d^7^4s^1^
CASSCF	0	25.64	24.64	68.99	76.72	0	74.79	50.72	94.44	76.74	81.77	92.68	104.27		25.89	42.31	64.08
C-CASPT2	0	22.37	24.27	59.50	64.34	0	55.88	57.44	74.88	77.69	73.70	80.74	86.09	0	19.76	33.81	51.04
s-ccCA	0		22.98			0	49.39							0	20.29		
C-MRCI+Q	0	22.15	22.16	58.52	62.27	0	50.47	54.84	71.80	74.70	72.41	78.00		0	22.70	36.73	52.88
Expt.^a^	0	21.71	23.13	58.67	62.46	0	49.47	53.10	67.22	70.89	72.28	77.86	86.90	0	20.18	34.32	49.41
C-MRCI+Q-Expt.	0	0.44	–0.97	–0.15	–0.19	0	1.00	1.74	4.58	3.81	0.13	0.14		0	2.52	2.41	3.47
C-CASPT2-Expt.	0	0.66	1.14	0.83	1.88	0	6.41	4.34	7.66	6.80	1.42	2.88	–0.81	0	–0.42	–0.51	1.63
previous work^b^	0	–0.50^c^	–0.70^c^			0	–0.2^c^							0	2.3^c^	1.6^c^	
			0.31^d^				0.98^d^								1.35^d^		
			–0.12^e^				0.56^e^								0.55^e^		
			5.47^f^				2.42^f^								3.80^f^		
							1.96^g^								1.73^g^		

a J-averaged experimental energies from ref 83.

b Theor—Expt; from ref 83.

c MRCI+Q from ref 26.

d ACPF from ref 14.

e Peterson coupled cluster composite approach from ref 14.

f CASPT2 from ref 13.

g CCSD­(T) from ref 13.

h The energies were extrapolated using aug-cc-pwCVXZ-DK (X = T, Q, and 5).

**Table 3:** Relative Ground and Excitation Energies (in kcal mol^–1^) of Co, Ni, and Cu Elements with s-ccCA, CASSCF/CBS, C-CASPT2/CBS, and C-MRCI+Q/CBS Levels^l^

	Co					Ni							Cu				
	a^4^F	b^4^F	a^2^F	a^4^P	b^4^P	a^3^D	a^3^F	a^1^D	b^1^D	a^1^S	a^3^P	a^1^G	a^2^S	a^2^D	a^2^P	a^4^P	a^4^F
methods	3d^7^4s^2^	3d^8^4s^1^	3d^8^4s^1^	3d^7^4s^2^	3d^8^4s^1^	3d^9^4s^1^	3d^8^4s^2^	3d^9^4s^1^	3d^8^4s^2^	3d^10^	3d^8^4s^2^	3d^8^4s^2^	3d^10^4s^1^	3d^9^4s^2^	3d^10^4p^1^	3d^9^4s^1^4p^1^	3d^9^4s^1^4p^1^
CASSCF	0	19.93	32.96	45.22	62.32	0	–23.47	8.16	21.86	104.83^j^	25.96	43.58	0	–0.78	75.54	68.43	74.23
C-CASPT2	0	10.26	21.63	38.40	44.95	0	–0.68	9.58	34.18	39.10	40.62	59.53	0	27.42	94.14	108.38	113.16
s-ccCA	0	9.43				0	0.01			39.79			0	34.13	88.11	114.84	
C-MRCI+Q	0	11.77	22.38	39.80	46.15	0	–0.95	7.57	37.37	40.25	42.10	62.53	0	33.75	92.76	111.72	116.66
Expt.^a^	0	9.62	20.26	37.69	42.19	0	0.69	7.66	36.57	40.02	42.79	61.10	0	34.37	87.78	113.52	118.13
C-MRCI+Q-Expt.	0	2.15	2.12	2.11	3.96	0	–1.64	–0.09	0.80	0.23^k^	–0.69	1.43	0	–0.62	4.98	–1.80	–1.47
C-CASPT2-Expt.	0	0.64	1.37	0.71	2.76	0	–1.37	1.92	–2.39	–0.92^k^	–2.17	–1.57	0	–6.95	6.36	–5.14	–4.97
previous work^b^	0	0.56^c^	2.1^e^			0	–0.61^c^	1.4^e^	–3.13^g^	1.26^g^	–4.05^g^	–2.53^g^	0	–0.56^c^			
		1.63^d^					–2.10^d^	–1.20^g^		2.5^h^				–2.20^d^			
		0.2^e^					–3.0^e^	–0.30^i^		0.80^i^				–3.5^e^			
		1.68^f^					–1.15^f^							0.91^f^			

a J-averaged experimental energies from ref 83.

b Theor—Expt; from ref 83.

c Peterson coupled cluster composite approach from ref 14.

d ACPF from ref 14.

e MRCI+Q from ref 26.

f CCSD­(T) from ref 13.

g CASPT2 from ref 9.

h CASPT2 from ref 13.

i CASPT2 from ref 16.

j ^1^S excitation energy obtained from a state-average CASSCF.

k C-MRCI+Q and C-CASPT2 from state-specific calculations.

l The energies were extrapolated using aug-cc-pwCVXZ-DK (X = T, Q, and 5).

**Table 4:** Relative Ground and Excitation Energies (in kcal mol^–1^) of Zn and Cd Elements with s-ccCA, CASSCF/CBS, C-CASPT2/CBS, and C-MRCI+Q/CBS Levels^c^

	Zn						Cd					
	a^1^S	a^3^P	a^1^P	a^3^S	b^1^S	b^3^P	a^1^S	a^3^P	a^1^P	a^3^S	b^1^S	b^3^P
methods	3d^10^4s^2^	3d^10^4s^1^4p^1^	3d^10^4s^1^4p^1^	3d^10^4s^1^5s^1^	3d^10^4s^1^5s^1^	3d^10^4s^1^5p^1^	4d^10^5s^2^	4d^10^5s^1^5p^1^	4d^10^5s^1^5p^1^	4d^10^5s^1^6s^1^	4d^10^5s^1^6s^1^	4d^10^5s^1^6p^1^
CASSCF	0	78.79	121.58	135.56	143.16	158.29	0	72.55	112.46	126.90	133.47	147.69
C-CASPT2	0	92.42	134.37	152.21	156.19	174.39	0	90.86	128.23	148.87	152.01	169.43
s-ccCA	0			153.71			0	89.27				
C-MRCI+Q	0	92.46	134.60	152.68	158.65	175.47	0	87.56	125.30	145.22	150.37	166.15
Expt.^a^	0	93.48	133.65	153.46	159.51	175.27	0	89.34	124.92	147.20	152.42	167.40
C-MRCI+Q-Expt.	0	–1.02	0.95	–0.78	–0.86	0.20	0	–1.78	0.38	–1.98	–2.05	–1.25
C-CASPT2-Expt.	0	–1.06	0.72	–1.25	–3.32	–0.88	0	1.52	3.31	1.67	–0.41	2.02
previous work^b^	0						0					

a J-averaged experimental energies from ref 83.

b Theor—Expt; from ref 83.

c The energies were extrapolated using aug-cc-pwCVXZ-DK and aug-cc-pwCVXZ-PP (X = T, Q, and 5) for Zn and Cd, respectively.

Overall, the current results are consistent with those of other studies,^13,14,26^ suggesting that C-MRCI+Q and ACPF methods outperform C-CASPT2. C-CASPT2 was found here to overestimate the excitation energies and exhibit a reversal in the order of excited states (Table 2). Notably, the C-CASPT2 excitation energies reported herein are higher for the first excited states, which are well separated, as compared to previously reported C-CASPT2 calculations, which may be ascribed to including a^4^D and b^4^D excited states in the SA-CASSCF reference wave function.

Only the first three excited states of the Fe atom were examined due to state mixing of the subsequent states. A comparison of the current findings with those of prior studies confirms that C-MRCI+Q tends to overestimate the excitation energies, while ACPF yields a lower error. For example, the error was 2.52 and 1.35 kcal mol^–1^ for C-MRCI+Q/CBS and ACPF/CBS, respectively.^14^ This difference can be attributed to the incorporation of the 3s and 3p electron correlation in the latter, whereas the former considered only the 3s electron correlation, revealing the importance of the 3p correlation. However, C-CASPT2/CBS yields a low error for the three excited states of iron with a MAD of 0.85 kcal mol^–1^.^13^

Similarly, C-CASPT2 performed well for the first four excited states of cobalt, resulting in a MAD of 1.37 kcal mol^–1^. Only four excited states were considered because including a^2^G state in the SA-CASSCF reference wave function resulted in a destabilization of the first excited states due to state mixing.

The first six excited states of the Ni atom were probed, and a^1^G excited state was included to maintain the degeneracy of the ground and excited states. For instance, there was no degeneracy for the ground and excited states in the absence of a^1^G state, and a^3^D → a^1^S excitation energy exhibited an error of 16.99 kcal mol^–1^ at C-CASPT2/aug-cc-pwCV5Z-DK. Based upon the SA-CASSCF results, the a^1^S excited state had the highest excitation energy with an energy of 104.83 kcal mol^–1^ compared to the other singlet states sharing the same symmetry (see Table 3). Consequently, it was demanding to access this state with C-MRCI+Q and C-CASPT2; therefore, a^3^D and a^1^S states were computed separately (state-specific calculation) by employing the same active space and number of correlated electrons as in the SA-CASSCF calculations. Errors of 0.23 and −0.92 kcal mol^–1^ were found for the excited state a^1^S at C-MRCI+Q/CBS and C-CASPT2/CBS, respectively (see Table 3). It is worth mentioning that the error of a^1^S excited state was −0.19 kcal mol^–1^ at C-CASPT2/aug-cc-pwCVQZ-DK, aligning very well with the experimental energy. Overall, C-CASPT2 leads to predictions for the other excited states, with a max. error of −2.39 kcal mol^–1^ for the b^1^D excited state. Compared to C-CASPT2 results, C-MRCI+Q shows intriguing behavior, yielding a MAD of 0.81 kcal mol^–1^, even though only the 3s electrons are considered at C-MRCI+Q. On the other hand, Balabanov and Peterson reported an error of −2.10 kcal mol^–1^ for the a^3^F excited state at the ACPF/CBS level.^14^ According to the current results, it can be concluded that the best way to accurately calculate a^3^D →a^1^S excitation energy is for it to be computed separately (state specifically) due to the significant error of 64.81 kcal mol^–1^ with the SA-CASSCF calculation, which will unlikely be significantly improved using the C-MRCI+Q and C-CASPT2 methods.

The first four excited states for Cu were computed; calculating the b^2^S excited state required including the 5s orbital in the active space, which significantly increases the computation complexity at C-MRCI+Q and C-CASPT2. C-MRCI+Q/CBS predictions are closer to the experimental data for a^2^D, a^4^P, and a^4^F excited states with the exception of a ^2^P excited state, where a notable error of 4.98 kcal mol^–1^ was obtained. C-CASPT2/CBS resulted in significant errors with an MAD of 5.86 kcal mol^–1^ for all the excited states. Raab and Roos reported an error of 0.91 kcal mol^–1^ for a^2^D excited state using C-CASPT2/ANO-RCC.^13^ It is noteworthy to mention that the order of ground and excited states was reversed when SA-CASSCF was used, where a^2^D state was found to be the ground state, a^2^S was the first excited state instead, and a ^2^P showed the highest excitation energy, which may be the reason for this significant deviation at C-CASPT2.

Accounting for the 3s3p correlation of transition metals can be challenging for CASPT2, mainly when a large number of electrons is involved, such as for late 3d and 4d transition metals.^81^ Therefore, the CASPT2 + CCSD­(T) approach was utilized, where the 3s3p correlation was calculated using single reference coupled cluster methods. This approach is applicable if the investigated states can be represented by one leading determinant. Here, the valence correlation was determined using CASPT2, and the core–valence correlation (Δ*E*_CV_) was calculated using CCSD­(T)/aug-cc-pwCV5Z-DK. As illustrated in Table S8, the error in the computed excitation energies decreased, lowering the MAD from 5.86 to 3.25 kcal mol^–1^. For instance, the error in the a^2^D excited state was reduced from −6.95 to −3.47 kcal mol^–1^, indicating the effectiveness of the adopted approach in mitigating the core–valence correlation error. However, the a^2^P excitation energy did not exhibit any improvement. By including b^2^S, an error of only 3.28 instead of 6.58 kcal mol^–1^ was found, highlighting the role of the b^2^S excited state in stabilizing a^2^P excited state. Further, the order of the ground and excited states is more fully depicted by including the b^2^S excited state, with the ground state coming first, followed by the a^2^D,a^4^P,a^2^P, and a^4^F excited states (see Table S9).

Comparing the late transition metals, Zn exhibited the lowest MAD of 0.76 kcal mol^–1^ at C-MRCI+Q/CBS, even in the absence of the 3d orbitals in the active space (see Figure 1). Further, for C-CASPT2/CBS, an error of less than 1.25 kcal mol^–1^ was found for all of the investigated excited states except for the b^1^S excited state, which resulted in an error of −3.32 kcal mol^–1^.

Generally, the computed excitation energies using C-MRCI+Q and C-CASPT2 demonstrated similar MADs when extrapolated to the CBS limit for aug-cc-pwCVXZ-DK and aug-cc-pVXZ-DK (X = T, Q, or 5), with the exception of the excitation energies of Ni at C-CASPT2 (see Tables S1–S4). For instance, the MADs at extrapolated C-CASPT2/CBS for aug-cc-pwCVXZ-DK and aug-cc-pVXZ-DK (X = T, Q, and 5) were 1.72 and 2.18 kcal mol^–1^, respectively (see Figures 1 and S1). The error for the extrapolated a^3^F excitation energy was −2.86 kcal mol^–1^ for the aug-cc-pVXZ-DK energies, which is higher than the −1.37 kcal mol^–1^ observed for aug-cc-pwCVXZ-DK (X = T, Q, and 5) energies.

For the s-ccCA, a max. error of 1.32 kcal mol^–1^ for a^4^P excited state of Cu (see Figure 1) was obtained. These results agree well with those of other studies.^11,13,14^ A 1.92 kcal mol^–1^ error was observed for a^6^D excited state of the Mn atom. Interestingly, s-ccCA predicted a^3^D state to be the ground state for Ni; however, the a^3^F state was found to be the ground state with the MR methods (see Table 3); experimentally, the difference between the ground and first excited state of Ni is very small, 0.69 kcal mol^–1^.

For Ni, the contribution from relativistic effects, obtained as E­[CCSD­(T)-DKH]-E­[CCSD­(T)], to the composite energy resulted in the lowering of the excitation energy by ∼8.36 kcal mol^–1^ for a^3^D → a^3^F. The full triples, CCSDT-CCSD­(T) contribution, was the second largest contribution, reducing the excitation energy by −0.78 kcal mol^–1^. The CCSDTQ and CCSDTQP contributions were similar in size but opposite in sign and therefore canceled one another. For all of the 3d transition metals considered with s-ccCA, the relativistic correction had the largest impact on the composite scheme, although this should not be entirely surprising. It has been shown in the development of the 3d correlation consistent basis sets that scalar relativistic effects (relativistic electron velocity and Mass-Darwin contributions) become important, and nonrelativistic computations do not address these effects.^72^ CCSD­(T)-DKH captures most of these relativistic effects. In earlier studies of 3d species, s-ccCA using CCSD­(T)-DKH and the aug-cc-pVQZ-DK basis set has been effective.^60,62^

Overall, the importance of valence higher-order correlation beyond CCSD­(T) is in the order CCSDT > CCSDTQ > CCSDTQP. With the exception of the Ni, the contributions are additive, without contributions canceling one another. Ni sees for its a^3^D → a^3^F excitation a contribution from a higher-order correlation of −0.79 kcal mol^–1^, mostly from CCSDT-CCSD­(T); T and Q cancel. Without the full triple core–valence term, this led to an excitation energy of −0.09 kcal mol^–1^. This excitation energy would indicate that the a^3^F state had the lowest energy. Overall, CCSDT-CV is small but non-negligible for excitation energies, ranging between −0.55 kcal mol^–1^ for the a^1^Sstate of Ni to as small as −0.04 kcal mol^–1^ for the excitation energy from a^4^F → b^4^F for cobalt; for most states, the contribution is ∼±0.12 kcal mol^–1^ or less. Overall, although the energetic contributions for terms beyond CCSD­(T)/CBS+DKH are small, they are necessary for s-ccCA to obtain its accuracy. Taken together, these findings suggest that the s-ccCA demonstrates higher accuracy compared to MR methods for the states with a single reference character.

### 3.2 4d Transition Metals

As for the 3d transition metal atoms, C-MRCI+Q was found to outperform C-CASPT2, rendering a lower error for the first five excitation energies for early 4d transition metal atoms (Y–Mo). MADs of 0.74, 0.90, 0.39, and 0.47 kcal mol^–1^ were found for Y, Zr, Nb, and Mo with C-MRCI+Q, respectively, while higher MADs of 2.38, 1.00, 1.97, and 2.19 kcal mol^–1^ obtained using C-CASPT2 (see Tables 5 and 6 and Figure 2). Comparing early Y–Mo transition metal atoms, the Y atom exhibited the largest max. error of 6.02 kcal mol^–1^ for the a^2^P excited state at C-CASPT2/CBS. The error was noted to increase by extrapolation to the CBS limit. For instance, an error of 3.65 kcal mol^–1^ was obtained for the a^2^P excited state at C-CASPT2/aug-cc-pwCVTZ-PP, respectively, indicating that extrapolation to the CBS limit does not always reduce the difference from experiment. The same behavior was also noted for certain excited states of Nb and Mo atoms. Raab and Roos also calculated the a^4^F, a^5^F, a^4^F, and a^5^D excitation energies of Y, Zr, Nb, and Mo atoms at C-CASPT2/ANO-RCC with values of −0.89, −2.48, 3.13, and 2.38 kcal mol^–1^, respectively.^13^

**Table 5:** Relative Ground and Excitation Energies (in kcal mol^–1^) of Y, Zr, and Nb Elements with s-ccCA, CASSCF/CBS, C-CASPT2/CBS, and C-MRCI+Q/CBS Levels^f^

	Y						Zr						Nb					
	a^2^D	a^2^P	a^4^F	b^4^F	a^4^P	a^2^F	a^3^F	a^3^P	a^5^F	a^1^D	a^1^G	a^5^P	a^6^D	a^4^F	a^4^P	a^4^D	a^2^G	a^2^D
methods	4d^1^5s^2^	5s^2^5p^1^	4d^2^5s^1^	4d^1^5s^1^5p^1^	4d^2^5s^1^	4d^2^5s^1^	4d^2^5s^2^	4d^2^5s^2^	4d^3^5s^1^	4d^2^5s^1^	4d^2^5s^2^	4d^3^5s^1^	4d^4^5s^1^	4d^3^5s^2^	4d^3^5s^2^	4d^4^5s^1^	4d^3^5s^2^	4d^3^5s^2^
CASSCF	0	24.08	37.65	38.27	47.96	49.90	0	14.30	19.32	14.95	26.72	38.51	0	–0.41	15.37	28.65	25.53	30.62
C-CASPT2	0	36.80	30.25	44.80	46.08	44.76	0	12.06	12.41	12.24	22.29	29.96	0	5.36	16.62	24.97	26.44	30.18
s-ccCA	0		30.84				0		13.18				0	4.96				
C-MRCI+Q	0	30.24	31.56	44.23	44.51	45.03	0	11.42	14.16	11.02	21.16	30.55	0	3.35	14.23	24.80	24.01	27.05
Expt.^a^	0	30.78	31.33	43.95	43.08	43.79	0	10.09	13.55	12.52	20.97	29.68	0	4.25	14.14	24.19	24.22	26.92
C-MRCI+Q-Expt.	0	–0.54	0.23	0.28	1.43	1.24	0	1.33	0.61	–1.50	0.19	0.87	0	–0.89	0.09	0.61	–0.21	0.13
C-CASPT2-Expt.	0	6.02	–1.08	0.85	3.00	0.97	0	1.97	–1.14	–0.28	1.32	0.28	0	1.11	2.48	0.78	2.22	3.26
previous work^b^	0		0.2^c^				0		0.3^c^				0	0.1^d^				
			1.0^d^						1.0^d^					3.13^e^				
			–0.89^e^						–2.48^e^									

a J-averaged experimental energies from ref 83.

b Theor—Expt; from ref 83.

c Peterson coupled cluster composite approach from ref 31.

d CCSD­(T) from ref 13.

e CASPT2 from ref 13.

f The energies were extrapolated using aug-cc-pwCVXZ-PP (X = T, Q, and 5).

**Table 6:** Relative Ground and Excitation Energies (in kcal mol^–1^) of Mo, Tc, and Ru Elements with s-ccCA, CASSCF/CBS, C-CASPT2/CBS, and C-MRCI+Q/CBS Levels^f^

	Mo						Tc								Ru						
	a^7^S	a^5^S	a^5^D	a^5^G	a^5^P	b^5^D	a^6^S	a^6^D	a^4^D	a^4^P	a ^4^F	a ^4^G	a^8^P	a ^4^H	a^5^F	a^3^F	a^5^D	a^5^P	b^3^F	a^3^P	a^3^G
methods	4d^5^5s^1^	4d^5^5s^1^	4d^4^5s^2^	4d^5^5s^1^	4d^5^5s^1^	4d^5^5s^1^	4d^5^5s^2^	4d^6^5s^1^	4d^6^5s^1^	4d^6^5s^1^	4d^7^	4d^5^5s^2^	4d^6^5s^1^5p^1^	4d^6^5s^1^	4d^7^5s^1^	4d^7^5s^1^	4d^6^5s^2^	4d^7^5s^1^	4d^8^	4d^7^5s^1^	4d^7^5s^1^
CASSCF	0	36.55	35.94	59.76	65.67	70.80	0	22.89	49.61	58.55	67.06	60.68	47.77	69.78	0	21.80	15.06	30.12	37.46	36.39	39.60
C-CASPT2	0	31.34	37.36	49.59	55.48	60.10	0	10.18	33.29	44.03	46.99	50.53	54.48	49.68	0	18.45	21.58	23.85	27.97	30.16	34.08
s-ccCA	0		35.59				0	9.46							0		20.03				
C-MRCI+Q	0	31.16	33.14	48.11	53.38	57.95	0	11.39	34.92	43.33	49.55	48.78	51.33	51.22	0	17.96	19.38	23.74	25.62	27.97	36.17
Expt.^a^	0	30.79	33.83	47.88	52.38	58.02	0	9.37	31.54	39.45	53.78	46.26	48.70	48.67	0	18.04	20.00	20.93	25.18	27.90	32.65
C-MRCI+Q-Expt.	0	0.37	–0.69	0.23	1.00	–0.07	0	2.02	3.38	3.88	–4.23	2.52	2.63	2.55	0	–0.07	–0.62	2.81	0.44	0.07	3.52
C-CASPT2-Expt.	0	0.55	3.53	1.71	3.10	2.08	0	0.81	1.75	4.58	–6.79	4.27	5.78	1.01	0	0.41	1.58	2.92	2.79	2.26	1.43
previous work^b^	0		1.2^c^				0	0.4^c^							0		–0.3^c^				
			1.7^d^					4.0^d^									–4.1^d^				
			2.38^e^					3.78^e^									–2.01^e^				

a J-averaged experimental energies from ref 83.

b Theor—Expt; from ref 83.

c Peterson coupled cluster composite approach from ref 31.

d CCSD­(T) from ref 13.

e CASPT2 from ref 13.

f The energies were extrapolated using aug-cc-pwCVXZ-PP (X = T, Q, and 5).

For the Tc atom, the ground and excited states up to a^4^H state were investigated, with only one of two ^4^F excited states considered to maintain the degeneracy in the SA-CASSCF calculations. However, the largest CI coefficients in the CI vectors were found to represent two different electronic configurations for the ^4^F excited state; two determinants had both 5s and 4d orbitals occupied, and five determinants had only 4d orbitals occupied, indicating that a^4^F and b^4^F are mixed, with the main contribution from a^4^F excited state. Therefore, the *J*-averaged experimental energy of the ^4^F excited state was estimated to be 53.78 kcal mol^–1^ for a^4^F excited state. The findings, as shown in Table 6 and Figure 2, reveal that both C-MRCI+Q and C-CASPT2 result in large MADs of 3.03 and 3.57 kcal mol^–1^, with max. errors of −4.23 and −6.79 kcal mol^–1^, respectively. This substantial deviation from experimental data may be attributed to the mixing of low-lying states. Hence, calculating just the first three excited states of Tc, which are well-separated in energy, can lead to a lower MAD than experimental values. Raab and Roos computed a ^6^D excitation energy, giving an error of 3.78 kcal mol^–1^, which may be minimized by extrapolation to the CBS limit.^13^

The ground and first six excited states of Ru were computed, and a ^3^G excited state was considered in SA-CASSCF to keep the degeneracy and stabilize the a^3^F excited state. For instance, the error in the a^3^F excitation energy was reduced from 8.19 to 1.19 kcal mol^–1^ with MRCI+Q/aug-cc-pwCV5Z-PP by adding a^3^G excited state. Though there was a high density of states observed for Ru within a narrow energy range, the correct ordering of the ground and excited states was obtained with MADs of 0.67 and 1.66 kcal mol^–1^ at C-MRCI+Q/CBS and C-CASPT2/CBS, respectively (see Table 6). Interestingly, in contrast to what was found for the valence isoelectronic Fe, C-MRCI+Q for Ru gave a smaller error for the excitation energies compared to C-CASPT2.

For Rh, the ground and excited states up to the b^2^D state were investigated to maintain the electronic degeneracy. The excitation energies obtained from C-MRCI+Q/CBS calculations were generally consistent with the experimental data, except for the a^2^P and b^2^D excited states. The order of these states was reversed; the b^2^D state had a lower excitation energy than a^2^P state. Further, it was noted that the error in excitation energies increases when extrapolating to the CBS limit. Specifically, at C-MRCI+Q/aug-cc-pwCVTZ-PP, a MAD of 1.74 kcal mol^–1^ was obtained, while at C-MRCI+Q/CBS, the MAD reached 2.12 kcal mol^–1^. On the other hand, C-CASPT2/CBS exhibited a larger error, leading to a MAD of 2.55 kcal mol^–1^. Comparing the computed a^2^D excited state at C-CASPT2/CBS and C-CASPT2/ANO-RCC,^13^ the former rendering a larger error. Compared to the 4d transition metal atoms, C-MRCI+Q and C-CASPT2 demonstrated the largest MADs for Pd, with values of 5.08 and 4.37 kcal mol^–1^, respectively, along with a max. error of 10.95 and −6.67 kcal mol^–1^. It is worth mentioning that C-CASPT2 results in low excitation energies compared to C-MRCI+Q, especially for a^1^P and a^3^P excited states (see Table 7). Previously reported errors in the computed a^3^D and a^3^F excitation energies were −0.13 and −6.16 kcal mol^–1^ at C-CASPT2/ANO-RCC,^13^ respectively, compared to −7.66 and −12.93 kcal mol^–1^ found with C-CASPT2/aug-cc-pwCVTZ-PP in the current study.

**Table 7:** Relative Ground and Excitation Energies (in kcal mol^–1^) of Rh, Pd, and Ag Elements with s-ccCA, CASSCF/CBS, C-CASPT2/CBS, and C-MRCI+Q/CBS Levels^f^

	Rh							Pd						Ag			
	a^4^F	a^2^D	a^2^F	a^4^P	a^2^P	b^4^F	b^2^D	a^1^S	a^3^D	a^1^D	a^3^F	a^1^P	a^3^P	a^2^S	a^2^P	a^2^D	b^2^S
methods	4d^8^5s^1^	4d^9^	4d^8^5s^1^	4d^8^5s^1^	4d^8^5s^1^	4d^7^5s^2^	4d^8^5s^1^	4d^10^	4d^9^5s^1^	4d^9^5s^1^	4d^8^5s^2^	4d^9^5p^1^	4d^8^5s^2^	4d^10^5s^1^	4d^10^5p^1^	4d^9^5s^2^	4d^10^6s^1^
CASSCF	0	19.09	16.26	33.23	43.38	33.09	40.84	0	5.53	15.40	49.35	85.89	86.79	0	80.51	84.93	101.01
C-CASPT2	0	12.07	14.69	26.18	36.24	39.09	32.60	0	15.51	25.21	71.2	101.23	100.86	0	90.98	96.29	122.11
s-ccCA	0	7.94				36.78		0	21.85		76.43			0	86.32	90.25	
C-MRCI+Q	0	6.87	13.71	26.41	35.69	38.40	31.75	0	23.69	32.08	80.67	110.62	111.10	0	86.67	95.93	
Expt.^a^	0	7.89	14.58	24.00	31.87	37.42	35.38	0	20.19	31.77	77.87	99.67	103.24	0	86.25	91.58	121.67
C-MRCI+Q-Expt.	0	–1.02	–0.87	2.41	3.82	0.98	–3.63	0	3.50	0.31	2.80	10.95	7.86	0	0.42	4.35	
C-CASPT2-Expt.	0	4.18	0.11	2.18	4.37	1.67	–2.78	0	–4.68	–6.56	–6.67	1.56	–2.38	0	4.73	4.71	0.44
previous work^b^	0	3.90^c^				0.7^e^		0	–0.4^c^		–5.9^c^			0		–4.87^d^	
		3.87^d^							–0.13^d^		–6.61^d^					–1.1^e^	
											–1.3^e^						

a J-averaged experimental energies from ref 83.

b Theor—Expt; from ref 83.

c CCSD­(T) from ref 13.

d CASPT2 from ref 13.

e Peterson coupled cluster composite approach from ref 31.

f The energies were extrapolated using aug-cc-pwCVXZ-PP (X = T, Q, and 5).

For the Ag atom, the first a^2^P and a^2^D excited states were calculated at C-MRCI+Q/CBS, resulting in errors of 0.42 and 4.35 kcal mol^–1^, respectively (see Table 7). Interestingly, errors of −0.09 and 2.01 kcal mol^–1^ were found for a^2^P and a^2^D excited states at C-MRCI+Q/aug-cc-pwCVTZ-PP, rendering a lower MAD of 1.05 kcal mol^–1^ compared to 2.38 kcal mol^–1^ at C-MRCI+Q/CBS. On the other hand, the first three excited states were calculated at the CASPT2 level, and core–valence correlation (Δ*E*_CV_) was determined at the CCSD­(T)/CBS level only for the first two excited states because the b^2^S excited state was not accessible via CCSD­(T). Surprisingly, the b^2^S excited state was found to have the lowest error of 0.44 kcal mol^–1^, compared to 4.73 and 4.71 obtained for a^2^P and a^2^D excited states. Similarly, the a^2^P, a^2^D, and b^2^S excited states exhibited lower errors of 4.17, 2.33, and −0.18 kcal mol^–1^ at C-CASPT2/aug-cc-pwCVTZ-PP.

The first five excited states of the Cd atom were computed, demonstrating lower MADs of 1.49 and 1.79 kcal mol^–1^ at C-MRCI+Q/CBS and C-CASPT2/CBS, respectively, compared with the late transition metal atoms (Rh–Ag). Noteworthy, CASPT2/aug-cc-pwCVTZ-PP yielded the lowest error for the computed excited states, resulting in a reduced MAD of 1.05 kcal mol^–1^.

Comparing the error in the extrapolated excitation energies to the CBS limit utilizing aug-cc-pwCVXZ-PP and aug-cc-pVXZ-PP (X = T, Q, or 5), similar errors were obtained, except for Ru and Cd atoms (see Tables S4–S7). For example, MADs with values of 0.67 and 1.53 kcal mol^–1^ were obtained for the Ru atom at extrapolated C-MRCI+Q/CBS using aug-cc-pwCVXZ-PP and aug-cc-pVXZ-PP (X = T, Q, and 5), respectively. Further, for the Cd atom, the extrapolated C-CASPT2/CBS using aug-cc-pwCVXZ-PP reduced the MAD by 0.33 kcal mol^–1^ compared to the corresponding one extrapolated using aug-cc-pVXZ-PP (X = T, Q, and 5).

For the single reference coupled cluster-based s-ccCA, it is again noted that for states with substantial single reference character, the composite outperforms C-MRCI+Q. This should not be surprising as s-ccCA accounts for contributions including quintuple excitations via CCSDTQP, while C-MRCI+Q is limited to only singles, doubles, and some triple and quadruple excitations via the Davidson correction. In addition, only one state is considered per calculation, while state average calculations are used for MR calculations. Mo is the one exception to the excellent performance of the composite with a difference between theory and experiment of 1.76 kcal mol^–1^. The C-MRCI+Q energy has a difference of −0.69 kcal mol^–1^ from that in the experiment. It was noted in the work by Peterson and workers that the a^5^D excited state of molybdenum is MR in nature as compared to the a^7^S ground state.^31^ As shown in Table S15, the a^7^S state of Mo has the largest T2_max_ amplitude of the 4d species considered, as well as a large D_1_ diagnostic of 0.199. For the 4d species, the MR diagnostics are not this notable. Even for Pd, which differs from the experiment by 1.66 kcal mol^–1^, s-ccCA still performs better than C-MRCI+Q, which has a difference of 3.50 kcal mol^–1^ for the a^3^D state. Mo is the one exception where s-ccCA sees a comparatively large error and C-MRCI-Q outperforms it. Despite being limited to single reference states, s-ccCA will generally perform better than C-MRCI+Q. Compared to the 3d species, the effect of scalar relativity on the composite scheme is comparatively less; for 4d s-ccCA, pseudopotentials are used to treat the inner electrons. These capture much of the important relativistic effects missing from a nonrelativistic approach. For the 4d species, a DKH-PP difference measures the missing scalar relativistic effects of the valence orbitals. This also serves as a measure of the error introduced by using pseudopotentials in place of explicit basis functions for the core electrons. As has been shown, this effect is generally small, but it cannot be neglected for accurate work. The largest DKH contribution is for the a^1^S → a^3^F excitation in Pd with 1.65 kcal mol^–1^. Tc sees a non-negligible decrease in the contribution to a^6^S→ a^6^D with −1.42 kcal mol^–1^. Similarly, to the 3d species for the contributions beyond CCSD­(T), they do not cancel each other out and are either usually all the same sign or one is opposite sign but small in magnitude. The order of importance of the higher-order correlation is CCSDT > CCSDTQ > CCSDTQP (T, Q, P), like the 3d species. For example, for a^1^S →a^3^F in Pd, the E­[CCSDT/CBS]-E­[CCSD­(T)/CBS] value is −0.91 kcal mol^–1^, while the E­[CCSDTQ/TZ]-E­[CCSDT/TZ] value contributes 0.01 kcal mol^–1^. E­[CCSDTQP/DZ]-E­[CCSDTQ/DZ] then contributes −0.03 kcal mol^–1^; overall, the contribution to the excitation energy is −0.93 kcal mol^–1^ for a higher-order correlation. Even the opposite sign quadruples term is not large enough to cancel T + P or compensate it. For the T-CV contribution, it is still small but non-negligible for accurate work. Mo, the worst performer of the s-ccCA in this set, contributes to the a^7^S → a^5^D excitation energy of 0.52 kcal mol^–1^. For other species considered, it ranges from ±0.17 kcal mol^–1^. Rarely the core–valence CCSDT contribution (T-CV) cancels the contribution to excitation energy from a higher-order coupled cluster in the case of Ru where T-CV compensates T + Q + P to a contribution of −0.05 kcal mol^–1^. Otherwise, T-CV either increases the magnitude or, at most, mildly compensates for T + Q + P. For the 4d species, higher correlation is a small but still necessary contribution to the computational excitation energy. But for states with substantial MR character or fully MR ones, such as open-shell singlets, C-MRCI+Q will be the only way to obtain excitation energies.

## 4 Conclusions

The efficacy of the s-ccCA, C-MRCI+Q, and C-CASPT2 methods in calculating the excitation energies of 3d and 4d transition metal atoms was examined in the current study. The s-ccCA demonstrated remarkable agreement with experimental data, which can be attributed to its capability to include higher-level coupled cluster corrections (triple, quadruple, and quintuple excitations). Despite the contributions from higher-order correlation being relatively small (∼1 kcal mol^–1^ total), they are necessary for the high accuracy of the s-ccCA method. Generally, when C-MRCI+Q and C-CASPT2 excitation energies were compared, a lower error was obtained for C-MRCI+Q, especially for 4d transition metal atoms. C-CASPT2 resulted in a significant error in the computed excitation energies for certain late transition metals, which may be attributed to an inaccurate description of core–valence correlation, as discussed for the Cu atom. However, the lowest error for C-CASPT2 excitation energies was obtained for Fe and Co atoms, aligning well with the experiment. Generally, the error in the computed excitation energies was higher for 4d transition metal atoms than for 3d transition metal atoms. However, CASPT2 is less computationally intensive and allows for the inclusion of core correlation orbitals for almost all 3d and 4d transition metal atoms, which provide a crucial contribution toward accurate energetics. Generally, the augmented polarized-weight-core correlated consistent basis sets; aug-cc-pwCVXZ (X = T, Q, and 5), resulted in the lowest error for the excitation energies for most of the elements, especially for Ni and Ru atoms. Extrapolating the excitation energies to the CBS limit reduces the error for most of the investigated elements except for Ag and Rh.

Finding accurate routes to calculate multiple excited states, with different spins and energetics, is essential toward predicting accurate spectroscopic properties of 3d and 4d transition metal atoms. Herein, we offer a multitude of options that vary in computational cost and accuracy toward calculating multiple single- and MR ground and excited state spins for all 3d and 4d transition metal atoms.

## Supplementary Material



## Notes

Excitation energies of 3d and 4d transition metals calculated with aug-cc-pVXZ (X = T, Q, and 5) basis set, along with their MAD, MSD, and max. error, computed CASPT2 excitation energies of Cu, contribution of s-ccCA terms, and T1, D1, and T2 diagnostics (PDF)
