# Supplementary material for: Ground and Excited States of 3d and 4d Transition Metals: Computational Insight into Atomic Properties
Source: J Phys Chem A. 2025 Sep 22;129(39):9027–40. doi: 10.1021/acs.jpca.5c05650 (PMC12498498; doi:10.1021/acs.jpca.5c05650)
Supplement: Supplementary file 1 [file jp5c05650_si_001.pdf]

Supporting Information for:

Ground and Excited States of 3*d* and 4*d* Transition Metals: Computational Insight into Atomic Properties

Ebtisam M. Z. Telb, Nuno M. S. Almeida, Bradley K. Welch, and Angela K. Wilson*
Department of Chemistry, Michigan State University, East Lansing, Michigan 48864

Table S1: Relative ground and excitation energies (in kcal mol^{-1}) of Sc, Ti, and V elements with s-ccCA, CASSCF/CBS, C-CASPT2/CBS, and C-MRCI+Q/CBS levels. The energies were extrapolated using aug-cc-pVXZ-DK (X= T, Q, 5).

Methods	Sc						Ti						V					
	a^2D	a^4F	a^2F	b^4F	a^4D	b^2D	a^3F	a^5F	a^1D	$a\ ^3P$	$b\ ^3F$	$a\ ^1G$	$a\ ^4F$	$a\ ^6D$	$a\ ^4D$	$a\ ^4P$	$a\ ^2G$	$a\ ^2P$
	$3d^14s^2$	$3d^24s^1$	$3d^24s^1$	$3d^14s^14p^1$	$3d^14s^14p^1$	$3d^14s^14p^1$	$3d^24s^2$	$3d^34s^1$	$3d^24s^2$	$3d^24s^1$	$3d^34s^1$	$3d^24s^2$	$3d^34s^2$	$3d^44s^1$	$3d^44s^1$	$3d^34s^2$	$3d^34s^2$	$3d^34s^2$
**CASSCF**	0	42.22	52.36	40.43	39.59	39.06	0	28.45	25.51	29.19	44.58	39.86	0	16.56	37.62	33.82	36.06	47.32
**C-CASPT2**	0	32.04	43.43	45.07	45.41	45.13	0	19.54	21.88	25.65	34.21	36.02	0	7.34	25.78	28.42	31.19	40.04
**s-ccCA**	0	32.88	----	----	----	----	0	18.65	----	----	----	----	0	5.49	----	----	----	----
**C-MRCI+Q**	0	33.71	44.12	45.19	46.13	45.72	0	19.68	20.01	23.53	34.20	33.94	0	6.63	25.04	26.76	30.82	39.08
**Expt.**[a]	0	32.91	42.58	45.11	45.83	45.61	0	18.58	20.11	23.80	32.74	34.01	0	5.65	23.67	26.86	30.56	38.56
**C-MRCI+Q - Expt.**	0	0.80	1.54	0.08	0.30	0.11	0	1.10	-0.10	-0.27	1.46	-0.07	0	0.98	1.37	-0.10	0.26	0.52
**C-CASPT2 - Expt.**	0	-0.87	0.85	-0.04	-0.42	-0.48	0	0.96	1.77	1.85	1.47	2.01	0	1.69	2.11	1.56	0.63	1.48
**Prev. Work**[b]	0	0.33[c]	3.9[e]	-	-	-	0	0.34[c]	1.2[e]	0.2[e]	-	-	0	0.21[c]	-	-	-	-
		0.32[d]	2.0[f]	-	-	-		0.33[d]	-	-	-	-		0.32[d]	-	-	-	-
		2.5[e]	-	-	-	-		-1.2[e]	-	-	-	-		-4.04[g]	-	-	-	-
		2.3[f]	-	-	-	-		-3.13[g]	-	-	-	-		-	-	-	-	-

[a] J-averaged experimental energies from ref [1]

[b] Theor – Expt; from ref [1]

[c] Peterson coupled cluster composite approach from ref [2]

[d] ACPF values from ref [2]

[e] MRCI+Q from ref [3]

[f] CASPT2 from ref [4]

[g] CASPT2 from ref [5]

Table S2 - Relative ground and excitation energies (in kcal mol^{-1}) of Cr, Mn, and Fe elements with s-ccCA, CASSCF/CBS, C-CASPT2/CBS, and C-MRCI+Q/CBS levels. The energies were extrapolated using aug-cc-pVXZ-DK (X= T, Q, 5).

	**Cr**					**Mn**								**Fe**			
	a ^{7}S	a ^{5}S	a ^{5}D	a ^{5}G	a ^{5}P	a ^{6}S	a ^{6}D	a ^{8}P	a ^{4}D	a ^{6}P	a ^{4}G	a ^{4}P	b ^{4}D	a ^{5}D	a ^{5}F	a ^{3}F	a ^{5}P
	3d^{5}4s^1	3d^{5}4s^1	3d^{4}4s^2	3d^{5}4s^1	3d^{5}4s^1	3d^{5}4s^2	3d^{6}4s^1	3d^{5}4s^{1}4p^1	3d^{6}4s^1	3d^{5}4s4p^1	3d^{5}4s^2	3d^{5}4s^2	3d^{5}4s^2	3d^{6}4s^2	3d^{7}4s^1	3d^{7}4s^1	3d^{7}4s^1
**CASSCF**	0	25.63	24.64	68.98	76.71	0	74.79	50.72	94.44	76.74	81.77	92.68	104.27	0	25.89	42.32	64.08
**C-CASPT2**	0	22.43	24.15	59.71	64.60	0	56.42	57.37	75.17	77.69	73.86	80.95	86.51	0	20.04	34.13	51.27
**s-ccCA**	0	----	22.98	----	----	0	49.39	----	----	----	----	----	----	0	20.29	----	----
**C-MRCI+Q**	0	22.17	22.12	58.67	62.45	0	50.64	54.71	71.87	74.58	72.54	78.20	---	0	22.83	36.87	52.94
**Expt.[a]**	0	21.71	23.13	58.67	62.46	0	49.47	53.10	67.22	70.89	72.28	77.86	86.90	0	20.18	34.32	49.41
**C-MRCI+Q-Expt.**	0	0.46	-1.01	0.00	-0.01	0	1.17	1.61	4.65	3.69	0.26	0.34	--	0	2.65	2.55	3.53
**C-CASPT2-Expt.**	0	0.72	1.02	1.04	2.14	0	6.95	4.27	7.95	6.80	1.58	3.09	-0.39	0	-0.14	-0.19	1.86
**Prev. Work[b]**	0	-0.50[c]	-0.70[c]	-	-	0	-0.2[c]	-	-	-	-	-	-	0	2.3[c]	1.6[c]	-
		-	0.31[d]	-	-		0.98[d]	-	-	-	-	-	-		1.35[d]	-	-
		-	-0.12[e]	-	-		0.56[e]	-	-	-	-	-	-		0.55[e]	-	-
		-	5.47[f]	-	-		2.42[f]	-	-	-	-	-	-		3.80[f]	-	-
		-	-	-	-		1.96[g]	-	-	-	-	-	-		1.73[g]	-	-

[a] J-averaged experimental energies from ref [1]

[b] Theor – Expt; from ref [1]

[c] MRCI+Q from ref [3]

[d] ACPF from ref [2]

[e] Peterson coupled cluster composite approach from ref [2]

[f] CASPT2 from ref [5]

[g] CCSD(T) from ref [5]

Table S3 - Relative ground and excitation energies (in kcal mol^{-1}) of Co, Ni, and Cu elements with s-ccCA, CASSCF/CBS, C-CASPT2/CBS, and C-MRCI+Q/CBS levels. The energies were extrapolated using aug-cc-pVXZ-DK (X= T, Q, 5).

Methods	Co					Ni							Cu				
	a ^4F	b ^4F	a ^2F	a ^4P	b ^4P	a ^3D	a ^3F	a ^1D	b ^1D	a ^1S	a ^3P	a ^1G	a ^2S	a ^2D	a ^2P	a ^4P	a ^4F
	$3d^74s^2$	$3d^84s^1$	$3d^84s^1$	$3d^74s^2$	$3d^84s^1$	$3d^94s^1$	$3d^84s^2$	$3d^94s^1$	$3d^84s^2$	$3d^{10}$	$3d^84s^2$	$3d^84s^2$	$3d^{10}4s^1$	$3d^94s^2$	$3d^{10}4p^1$	$3d^94s^14p^1$	$3d^94s^14p^1$
**CASSCF**	0	19.93	32.97	45.22	62.32	0	-23.47	8.16	21.86	104.83^j	25.96	43.58	0	-0.78	75.54	68.43	74.23
**C-CASPT2**	0	10.48	21.88	38.33	45.11	0	-2.17	9.21	33.72	39.07	40.00	59.03	0	27.32	94.07	108.14	112.92
**s-ccCA**	0	9.43	----	----	----	0	0.10	----	----	39.79	----	----	0	34.13	88.11	114.84	----
**C-MRCI+Q**	0	11.84	22.47	39.73	46.16	0	-0.96	7.59	37.41	40.27	42.02	62.58	0	33.71	92.74	111.70	116.64
**Expt.^{a}**	0	9.62	20.26	37.69	42.19	0	0.69	7.66	36.57	40.02	42.79	61.10	0	34.37	87.78	113.52	118.13
**C-MRCI+Q-Expt.**	0	2.22	2.21	2.04	3.97	0	-1.65	-0.07	0.84	0.25^k	-0.77	1.48	0	-0.66	4.96	-1.82	-1.49
**C-CASPT2-Expt.**	0	0.86	1.62	0.64	2.92	0	-2.86	1.55	-2.85	-0.95^k	-2.79	-2.07	0	-7.05	6.29	-5.38	-5.21
**Prev. Work ^{b}**	0	0.56^c	2.1^e	-	-	0	-0.61^c	1.4^e	-3.13^g	1.26^g	-4.05^g	-2.53^g	0	-0.56^c	-	-	-
		1.63^d	-	-	-		-2.10^d	-1.20^g	-	2.5^h	-	-		-2.20^d	-	-	-
		0.2^e	-	-	-		-3.0^e	-0.30^i	-	0.80^i	-	-		-3.5^e	-	-	-
		1.68^f	-	-	-		-1.15^f	-	-	-	-	-		0.91^f	-	-	-

[a] J-averaged experimental energies from ref [1]
[b] Theor – Expt; from ref [1]
[c] Peterson coupled cluster composite approach from ref [2]
[d] ACPF from ref [2]
[e] MRCI+Q from ref [3]
[f] CCSD(T) from ref [5]
[g] CASPT2 from ref [6]
[h] CASPT2 from ref [5]
[i] CASPT2 from ref [7]
[j] ^1S excitation energy obtained from a state-average CASSCF
[k] C-MRCI+Q and C-CASPT2 from state-specific calculations

Table S4 - Relative ground and excitation energies (in kcal mol^{-1}) of Zn and Cd elements with s-ccCA, CASSCF/CBS, C-CASPT2/CBS, and C-MRCI+Q/CBS levels. The energies were extrapolated using aug-cc-pVXZ-DK and aug-cc-pVXZ-PP (X= T, Q, 5) for Zn and Cd, respectively.

Methods	Zn						Cd					
	a ^1S	a ^3P	a ^1P	a ^3S	b ^1S	b ^3P	a ^1S	a ^3P	a ^1P	a ^3S	b ^1S	b ^3P
	$3d^{10}4s^2$	$3d^{10}4s^14p^1$	$3d^{10}4s^14p^1$	$3d^{10}4s^15s^1$	$3d^{10}4s^15s^1$	$3d^{10}4s^15p^1$	$4d^{10}5s^2$	$4d^{10}5s^15p^1$	$4d^{10}5s^15p^1$	$4d^{10}5s^16s^1$	$4d^{10}5s^16s^1$	$4d^{10}5s^16p^1$
**CASSCF**	0	78.79	121.58	135.56	143.16	158.29	0	72.42	112.39	127.78	133.60	147.60
**C-CASPT2**	0	92.37	134.30	152.16	156.14	174.32	0	90.90	128.34	150.25	152.85	169.55
**s-ccCA**	0	----	----	153.71	----	----	0	89.27	----	----	----	----
**C-MRCI+Q**	0	92.48	134.62	152.74	158.70	175.51	0	87.53	125.30	146.49	150.95	166.18
**Expt.**[a]	0	93.48	133.65	153.46	159.51	175.27	0	89.34	124.92	147.20	152.42	167.40
**C-MRCI+Q-Expt.**	0	-1.00	0.97	-0.72	-0.81	0.24	0	-1.81	0.38	-0.71	-1.47	-1.22
**C-CASPT2-Expt.**	0	-1.11	0.65	-1.30	-3.37	-0.95	0	1.56	3.42	3.05	0.43	2.15
**Prev. Work** [b]	0	-	-	-	-	-	0	-	-	-	-	-
		-	-	-	-	-		-	-	-	-	-

[a]J-averaged experimental energies from ref [1]

[b] Theor – Expt; from ref [1]

Table S5 - Relative ground and excitation energies (in kcal mol^{-1}) of Y, Zr, and Nb elements with s-ccCA, CASSCF/CBS, C-CASPT2/CBS, and C-MRCI+Q/CBS levels. The energies were extrapolated using aug-cc-pVXZ-PP (X= T, Q, 5).

Methods	Y						Zr						Nb					
	a ^2D	a ^2P	a ^4F	b ^4F	a^4P	a ^2F	a ^3F	a ^3P	a ^5F	a ^1D	a ^1G	a ^5P	a^6D	a^4F	a^4P	a^4D	a^2G	a^2D
	$4d^15s^2$	$5s^25p^1$	$4d^25s^1$	$4d^15s^15p^1$	$4d^25s^1$	$4d^25s^1$	$4d^25s^2$	$4d^25s^2$	$4d^35s^1$	$4d^25s^1$	$4d^25s^2$	$4d^35s^1$	$4d^45s^1$	$4d^35s^2$	$4d^35s^2$	$4d^45s^1$	$4d^35s^2$	$4d^35s^2$
**CASSCF**	0	24.08	37.65	38.27	47.96	49.9	0	14.3	19.32	14.95	26.72	38.51	0	-0.41	15.37	28.65	25.53	30.62
**C-CASPT2**	0	36.46	30.48	44.81	46.14	44.98	0	12.09	12.56	12.32	22.39	30.07	0	5.26	16.58	25.05	26.47	30.22
**s-ccCA**	0	----	30.84	----	----	----	0	----	13.18	----	----	----	0	4.96	----	----	----	----
**C-MRCI+Q**	0	30.00	31.75	44.28	44.61	45.22	0	11.47	14.23	11.09	21.26	30.61	0	3.30	14.24	24.81	24.07	27.12
**Expt.[a]**	0	30.78	31.33	43.95	43.08	43.79	0	10.09	13.55	12.52	20.97	29.68	0	4.25	14.14	24.19	24.22	26.92
**C-MRCI+Q-Expt.**	0	-0.78	0.42	0.33	1.53	1.43	0	1.38	0.68	-1.43	0.29	0.93	0	-0.95	0.10	0.62	-0.15	0.20
**C-CASPT2-Expt.**	0	5.68	-0.85	0.86	3.06	1.19	0	2.00	-0.99	-0.20	1.42	0.39	0	1.01	2.44	0.86	2.25	3.30
**Prev. Work [b]**	0	-	0.2[c]	-	-	-	0	-	0.3 [c]	-	-	-	0	0.1[d]	-	-	-	-
		-	1.0[d]	-	-	-		-	1.0[d]	-	-	-		3.13[e]	-	-	-	-
		-	-0.89[e]	-	-	-		-	-2.48[e]	-	-	-		-	-	-	-	-

[a] J-averaged experimental energies from ref [1]

[b] Theor – Expt; from ref [1]

[c] Peterson coupled cluster composite approach from ref [8]

[d] CCSD(T) from ref [5]

[e] CASPT2 from ref [5]

Table S6- Relative ground and excitation energies (in kcal mol^{-1}) of Mo, Tc, and Ru elements with s-ccCA, CASSCF/CBS, C-CASPT2/CBS, and C-MRCI+Q/CBS levels. The energies were extrapolated using aug-cc-pVXZ-PP (X= T, Q, 5).

Methods	Mo						Tc								Ru						
	a ^{7}S	a ^{5}S	a ^{5}D	a ^{5}G	a ^{5}P	b ^{5}D	a ^{6}S	a ^{6}D	a ^{4}D	a^{4}P	a ^{4}F	a ^{4}G	a^{8}P	a ^{4}H	a ^{5}F	a ^{3}F	a ^{5}D	a ^{5}P	b ^{3}F	a ^{3}P	a ^{3}G
	$4d^55s^1$	$4d^55s^1$	$4d^45s^2$	$4d^55s^1$	$4d^55s^1$	$4d^55s^1$	$4d^55s^2$	$4d^65s^1$	$4d^65s^1$	$4d^65s^1$	$4d^7$	$4d^55s^2$	$4d^65s^15p^1$	$4d^65s^1$	$4d^75s^1$	$4d^75s^1$	$4d^65s^2$	$4d^75s^1$	$4d^8$	$4d^75s^1$	$4d^75s^1$
**CASSCF**	0	36.55	35.94	59.76	65.67	70.80	0	22.89	49.61	58.55	67.06	60.68	47.77	69.78	0	21.80	15.06	30.12	37.46	36.39	39.60
**C-CASPT2**	0	31.42	37.31	49.76	55.67	60.24	0	10.42	33.57	44.35	47.37	50.74	54.43	50.07	0	18.48	21.37	23.82	25.76	30.21	34.18
**s-ccCA**	0	----	35.59	----	----	----	0	9.46	----	----	----	----	----	----	0	----	20.03	----	----	----	----
**C-MRCI+Q**	0	31.17	33.11	48.22	53.52	58.01	0	11.58	35.12	43.58	49.71	48.99	51.28	51.52	0	18.28	19.81	24.16	26.24	28.61	36.39
**Expt.[a]**	0	30.79	33.83	47.88	52.38	58.02	0	9.37	31.54	39.45	53.78	46.26	48.70	48.67	0	18.04	20.00	20.93	25.18	27.90	32.65
**C-MRCI+Q-Expt.**	0	0.38	-0.72	0.34	1.14	-0.01	0	2.21	3.58	4.13	-4.07	2.73	2.58	2.85	0	0.24	-0.19	3.23	1.06	0.71	3.74
**C-CASPT2-Expt.**	0	0.63	3.48	1.88	3.29	2.22	0	1.05	2.03	4.90	-6.41	4.48	5.73	1.40	0	0.44	1.37	2.89	0.58	2.31	1.53
**Prev. Work [b]**	0	-	1.2[c]	-	-	-	0	0.4[c]	-	-	-	-	-	-	0	-	-0.3[c]	-	-	-	-
		-	1.7[d]	-	-	-		4.0[d]	-	-	-	-	-	-		-	-4.1[d]	-	-	-	-
		-	2.38[e]	-	-	-		3.78[e]	-	-	-	-	-	-		-	-2.01[e]	-	-	-	-

[a]J-averaged experimental energies from ref [1]

[b] Theor – Expt; from ref [1]

[c] Peterson coupled cluster composite approach from ref [8]

[d] CCSD(T) from ref [5]

[e] CASPT2 from ref [5]

Table S7 - Relative ground and excitation energies (in kcal mol^{-1}) of Rh, Pd, and Ag elements with s-ccCA, CASSCF/CBS, C-CASPT2/CBS, and C-MRCI+Q/CBS levels. The energies were extrapolated using aug-cc-pVXZ-PP (X= T, Q, 5).

Methods	Rh							Pd						Ag			
	a ^4F	a ^2D	a ^2F	a ^4P	a ^2P	b^4F	b ^2D	a ^1S	a ^3D	a ^1D	a ^3F	a ^1P	a ^3P	a ^2S	a ^2P	a ^2D	b ^2S
	$4d^85s^1$	$4d^9$	$4d^85s^1$	$4d^85s^1$	$4d^85s^1$	$4d^75s^2$	$4d^85s^1$	$4d^{10}$	$4d^95s^1$	$4d^95s^1$	$4d^85s^2$	$4d^95p^1$	$4d^85s^2$	$4d^{10}5s^1$	$4d^{10}5p^1$	$4d^95s^2$	$4d^{10}6s^1$
**CASSCF**	0	19.09	16.26	33.23	43.38	33.09	40.84	0	5.53	15.4	49.35	85.89	86.79	0	80.51	84.93	101.01
**C-CASPT2**	0	12.15	14.71	26.14	36.22	38.91	32.67	0	15.40	25.11	70.93	101.03	100.57	0	91.00	96.26	122.20
**s-ccCA**	0	7.94	----	----	----	36.78		0	21.85	----	76.43	----	----	0	86.32	90.25	----
**C-MRCI+Q**	0	6.92	13.71	26.37	35.67	38.32	31.77	0	23.64	32.02	80.53	110.51	110.92	0	86.67	95.87	----
**Expt.**[a]	0	7.89	14.58	24	31.87	37.42	35.38	0	20.19	31.77	77.87	99.67	103.24	0	86.25	91.58	121.67
**C-MRCI+Q-Expt.**	0	-0.97	-0.87	2.37	3.80	0.90	-3.61	0	3.45	0.25	2.66	10.84	7.68	0	0.42	4.29	----
**C-CASPT2-Expt.**	0	4.26	0.13	2.14	4.35	1.49	-2.71	0	-4.79	-6.66	-6.94	1.36	-2.67	0	4.75	4.68	0.53
**Prev. Work** [b]	0	3.90[c]	-	-	-	0.7[e]	-	0	-0.4[c]	-	-5.9[c]	-	-	0	-	-4.87[d]	-
		3.87[d]	-	-	-	-	-		-0.13[d]	-	-6.61[d]	-	-		-	-1.1[e]	-
		-	-	-	-	-	-			-	-1.3[e]	-	-		-	-	-

[a]J-averaged experimental energies from ref [1]

[b] Theor – Expt; from ref [1]

[c] CCSD(T) from ref [5]

[d] CASPT2 from ref 13

[e] Peterson coupled cluster composite approach from ref [8]

Table S8: Computed CASPT2/CBS+ΔE_{CV} excitation energies of Cu compared to *J*-averaged experimental energies. All values in kcal mol^{-1}.

Methods	Cu				
	**a ^{2}S $3d^{10}4s^1$**	**a ^{2}D $3d^94s^2$**	**a ^{2}P $3d^{10}4p^1$**	**a ^{4}P $3d^94s^14p^1$**	**a ^{4}F^b $3d^94s^14p^1$**
**CASSCF**	**0**	-0.78	75.54	68.43	74.23
**CASPT2+ΔE_{CV}^{a}**	**0**	30.90	94.36	111.93	116.75
**Expt.**	**0**	34.37	87.78	113.52	118.13
**Theor.-Expt**	**0**	**-3.47**	**6.58**	**-1.59**	**-1.38**

^{a}ΔE_{CV} was calculated at CCSD(T)/aug-cc-pwCV5Z-DK
^{b}ΔE_{CV} for a ^{4}F was the same as for a ^{4}P

Table S9: The computed CASPT2/aug-cc-pwCV5Z-DK+ΔE_{CV} excitation energies of Cu in the presence of b ^{2}S excited state compared to *J*-averaged experimental energies. All values in kcal mol^{-1}.

Methods	Cu				
	**a ^{2}S $3d^{10}4s^1$**	**a ^{2}D $3d^94s^2$**	**a ^{2}P $3d^{10}4p^1$**	**a ^{4}P $3d^94s^14p^1$**	**a ^{4}F $3d^94s^14p^1$**
**CASSCF**	**0**	4.08	78.89	73.54	79.36
**CASPT2+ΔE_{CV}^{a}**	**0**	29.80	91.06	109.88	114.82
**Expt.**	**0**	34.37	87.78	113.52	118.13
**Theor.-Expt**	**0**	-4.57	3.28	-3.64	-3.31

^{a}ΔE_{CV} was calculated at CCSD(T)/aug-cc-pwCV5Z-DK

TABLE S10- s-ccCA excitation energies and the individual contributions to the s-ccCA composite energy for Sc-Co. All energies are in kcal mol^{-1}

Contributions	Sc($^2D \rightarrow ^4F$)	Ti($a^3F \rightarrow a^5F$)	V($a^4F \rightarrow a^6D$)	Cr($^7S \rightarrow ^5D$)	Mn($a^6S \rightarrow a^6D$)	Fe($a^5D \rightarrow a^5F$)	Co($a^4F \rightarrow b^4F$)
AWQZ	30.92	15.97	2.26	27.44	46.76	16.04	4.13
AW5Z	30.54	15.62	1.93	27.77	45.97	15.23	3.42
CBS	30.01	15.15	1.47	28.21	44.90	14.13	2.45
DKH	2.70	3.30	3.93	-4.79	4.61	5.89	6.97
T-TZ	0.27	0.23	0.26	-0.28	-0.07	0.12	0.25
T-QZ	0.25	0.20	0.23	-0.27	-0.02	0.20	0.37
CBS	0.22	0.16	0.20	-0.26	0.03	0.27	0.49
Q-TZ	0.00	0.03	0.17	-0.05	-0.08	-0.04	-0.06
P-DZ	0.00	0.00	0.14	0.00	0.01	0.03	0.06
CV-TZ	-0.05	0.01	0.11	-0.12	0.08	0.01	-0.04
Exc	32.88	18.65	6.04	22.98	49.55	20.29	9.87

Table S11 - s-ccCA excitation energies and the individual contributions to the s-ccCA composite energy for Ni-Zn. All energies are in kcal mol^{-1}

Contributions	Ni(a^{3}D → a^{3}F)	Ni(a^{3}D→^{1}S)	Cu(a^{2}S → a^{2}D)	Cu(a^{2}S → a^{2}P)	Cu(a^{2}S → a^{4}P)	Zn(a^{1}S → a^{3}S)
AWQZ	7.57	35.28	43.08	84.75	121.77	149.55
AW5Z	8.21	34.74	43.73	84.82	122.50	149.73
CBS	9.08	34.00	44.61	84.91	123.50	149.98
DKH	-8.38	5.17	-9.68	3.93	-6.04	3.97
T-TZ	-0.42	1.38	-0.64	-0.65	-1.97	-0.14
T-QZ	-0.59	1.62	-0.79	-0.60	-1.92	-0.18
CBS	-0.78	1.88	-0.95	-0.55	-1.87	-0.23
Q-TZ	0.06	-0.92	0.06	0.02	-0.62	0.04
P-DZ	-0.07	0.22	-0.10	0.03	-0.12	-0.02
CV-TZ	0.10	-0.55	0.18	-0.22	0.00	-0.03
Exc	0.01	39.79	34.13	88.11	114.84	153.71

Table S12- s-ccCA excitation energies and the individual contributions to the s-ccCA composite energy for Y-Ru. All energies are in kcal mol^{-1}

Contributions	Y(^{2}D → ^{4}F)	Zr(^{3}F → ^{5}F)	Nb(^{6}D → 4F)	Mo(^{7}S → ^{5}D)	Tc(^{6}S → ^{6}D)	Ru(^{5}F → ^{5}D)
AWQZ	31.13	13.24	5.29	36.53	13.00	16.50
AW5Z	30.82	12.92	5.63	36.83	12.04	17.52
CBS	30.40	12.49	6.10	37.23	10.73	18.94
DKH	0.22	0.27	-0.40	-0.74	-1.42	1.14
T-TZ	0.46	0.59	-0.91	-1.30	0.03	-0.05
T-QZ	0.42	0.55	-0.86	-1.18	0.03	-0.06
CBS	0.39	0.50	-0.81	-1.04	0.02	-0.07
Q-TZ	0.00	0.10	0.01	-0.36	0.06	-0.08
P-DZ	0.00	0.00	0.00	-0.01	0.00	-0.01
CV-TZ	-0.16	-0.18	0.05	0.52	0.07	0.10
Exc	30.84	13.18	4.96	35.59	9.46	20.03

Table S13- s-ccCA excitation energies and the individual contributions to the s-ccCA composite energy for Rh-Cd. All energies are in kcal mol^{-1}

Contributions	Rh($^4F \rightarrow {}^2D$)	Rh($^4F \rightarrow {}^4F$)	Pd($^1S \rightarrow {}^3D$)	Pd($^1S \rightarrow {}^3F$)	Ag($^2S \rightarrow 2P$)	Ag($^2S \rightarrow {}^2D$)	Cd($^1S \rightarrow {}^3P$)
AWQZ	11.55	33.83	20.32	72.18	86.77	87.87	89.47
AW5Z	11.01	34.72	20.96	73.60	86.81	88.66	89.53
CBS	10.28	35.94	21.85	75.54	86.86	89.74	89.61
DKH	-0.66	1.01	0.46	1.65	-0.12	0.81	0.03
T-TZ	-1.15	-0.08	-0.33	-0.46	-0.22	-0.13	-0.28
T-QZ	-1.01	-0.11	-0.48	-0.68	-0.27	-0.19	-0.39
CBS	-0.88	-0.15	-0.64	-0.91	-0.33	-0.26	-0.50
Q-TZ	-0.70	-0.08	0.10	0.02	0.02	-0.10	0.15
P-DZ	0.04	-0.01	-0.02	-0.03	0.00	-0.01	0.00
CV-TZ	-0.14	0.06	0.11	0.17	-0.11	0.06	-0.02
Exc	7.94	36.78	21.85	76.43	86.32	90.25	89.27

TABLE S14-Multireference diagnostics for Sc-Zn. $|T2_{max}|$ is the largest absolute doubles amplitude. – in a column means that the doubles amplitude was below the 0.05 cutoff.

Diagnostic	Sc ^{2}D	Sc ^{4}F	Ti a^{3}F	Ti a^{5}F	V a^{4}F	V a^{6}D	Cr ^{7}S	Cr ^{5}D
T_1	0.021	0.013	0.021	0.015	0.020	0.016	0.016	0.021
D_1	0.069	0.042	0.069	0.044	0.069	0.044	0.045	0.074
$\|T2_{max}\|$	0.050	-	0.051	-	0.052	-	-	0.055
Diagnostic	Mn a^{6}S	Mn a^{6}D	Fe a^{5}D	Fe a^{5}F	Co a^{4}F	Co b^{4}F		
T_1	0.019	0.020	0.020	0.020	0.019	0.021		
D_1	0.067	0.051	0.068	0.054	0.067	0.056		
$\|T2_{max}\|$	0.052	-	0.053	-	0.052	-		
Diagnostic	Ni a^{3}D	Ni a^{3}F	Ni a^{1}S	Cu a^{2}S	Cu a^{2}P	Cu a^{4}P	Zn a^{1}S	Zn a^{3}S
T_1	0.021	0.019	0.024	0.021	0.023	0.013	0.018	0.014
D_1	0.059	0.066	0.046	0.060	0.079	0.036	0.064	0.047
$\|T2_{max}\|$	-	0.053	-	-	-	-	-	-

TABLE S15-Multireference diagnostics for Y-Cd. $|T2_{max}|$ is the largest absolute doubles amplitude. – in a column means that the doubles amplitude was below the 0.05 cutoff.

Diagnostic	Y ^{2}D	Y ^{4}F	Zr ^{3}F	Zr ^{5}F	Nb ^{6}D	Nb ^{4}F	Mo ^{7}S	Mo ^{5}D
T_1	0.023	0.015	0.023	0.013	0.012	0.022	0.082	0.073
D_1	0.075	0.042	0.077	0.042	0.036	0.078	0.199	0.273
$\|T2_{max}\|$	-	-	-	-	0.080	-	0.108	0.052
Diagnostic	Tc ^{6}S	Tc ^{6}D	Ru ^{5}F	Ru ^{5}D	Rh a^{4}F	Rh ^{2}D	Rh b^{4}F	
T_1	0.020	0.014	0.015	0.020	0.020	0.050	0.020	
D_1	0.077	0.050	0.055	0.077	0.078	0.205	0.077	
$\|T2_{max}\|$	-	-	-	-	-	-	-	
Diagnostic	Pd ^{1}S	Pd ^{3}D	Pd ^{3}F	Ag ^{2}S	Ag ^{2}P	Ag ^{2}D	Cd ^{1}S	Cd ^{3}P
T_1	0.007	0.017	0.019	0.017	0.023	0.019	0.018	0.013
D_1	0.013	0.064	0.078	0.068	0.097	0.077	0.077	0.048
$\|T2_{max}\|$	-	-	-	-	-	-	-	-

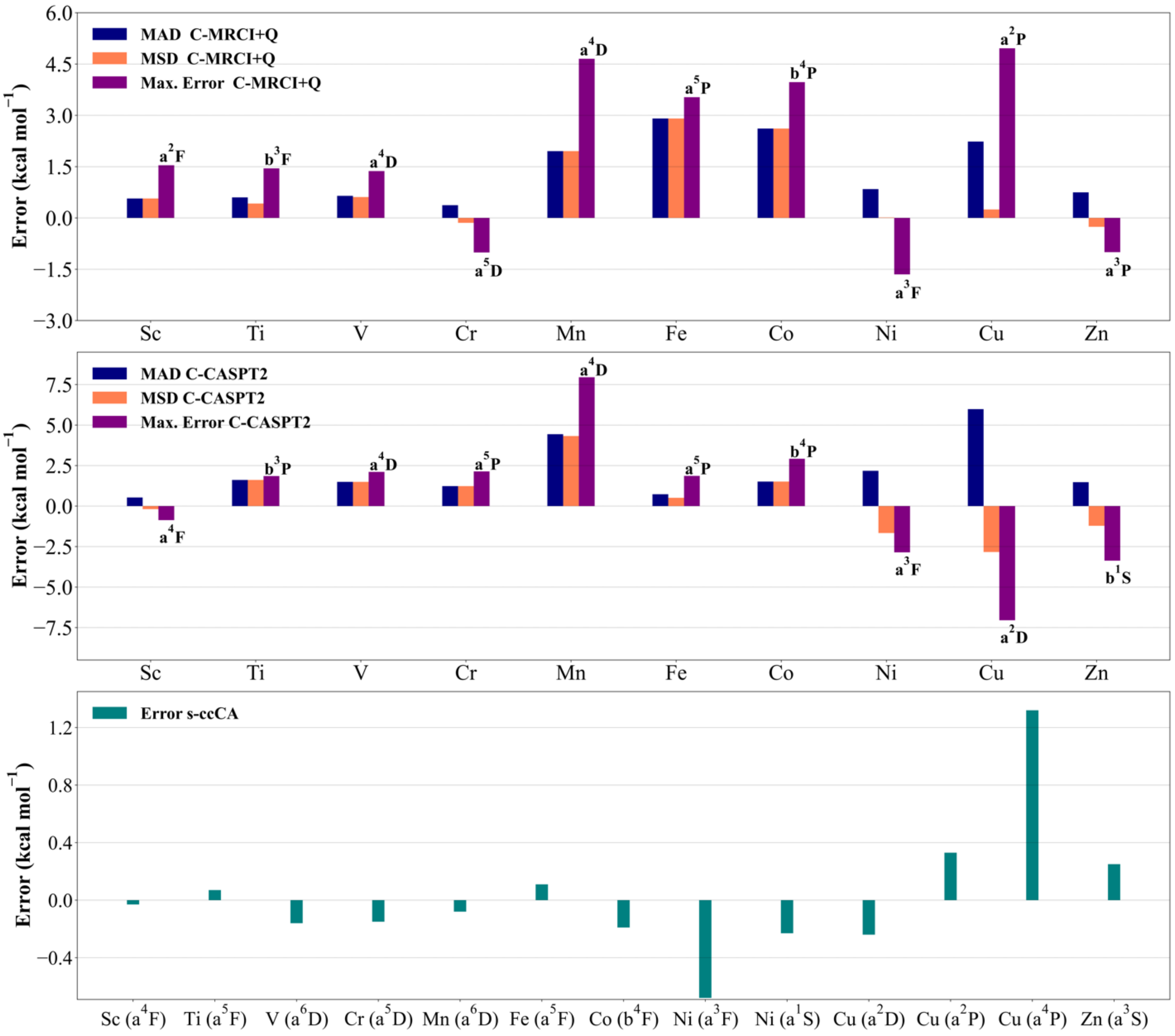

Fig. S1: The MAD, MSD, and Max. Error of 3*d* transition metal excitation energies calculated at C-MRCI+Q/CBS and CASPT2/CBS, and the error in the computed excitation energies using the s-ccCA composite approach

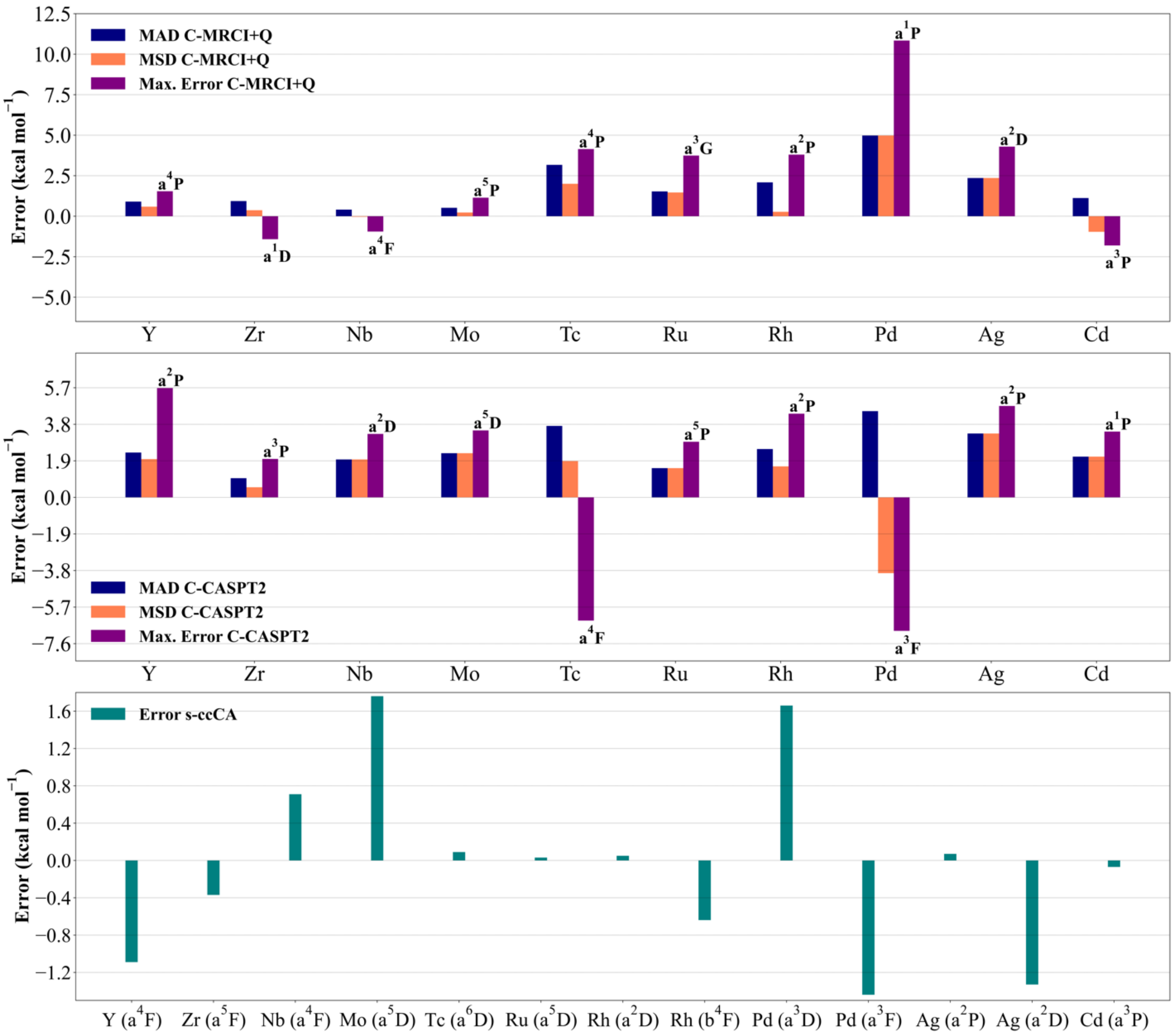

Fig. S2: The MAD, MSD, and Max. Error of 4*d* transition metal excitation energies calculated at C-MRCI+Q/CBS and CASPT2/CBS, and the error in the computed excitation energies using the s-ccCA composite approach.

References

(1) Kramida, A.; Ralchenko, Y.; Reader, J.; NIST ASD Team (2024. *NIST Atomic Spectra Database (ver. 5.11). Available:https://physics.nist.gov/asd [2025, June 9]*. https://doi.org/10.18434/T4W30F.

(2) Balabanov, N. B.; Peterson, K. A. Basis Set Limit Electronic Excitation Energies, Ionization Potentials, and Electron Affinities for the 3d Transition Metal Atoms: Coupled Cluster and Multireference Methods. *Journal of Chemical Physics* **2006**, *125* (7), 074110. https://doi.org/10.1063/1.2335444.

(3) Alizadeh Sanati, D.; Andrae, D. Low-Lying Electronic Terms of Diatomic Molecules AB (A = Sc–Ni, B = Cu/Ag/Au). *Mol Phys* **2020**, *118* (21–22), 1772514. https://doi.org/10.1080/00268976.2020.1772514.

(4) Lodi, L.; Yurchenko, S. N.; Tennyson, J. The Calculated Rovibronic Spectrum of Scandium Hydride, ScH. *Mol Phys* **2015**, *113* (13–14), 1998–2011. https://doi.org/10.1080/00268976.2015.1029996.

(5) Raab, J.; Roos, B. O. Excitation Energies for Transition Metal Atoms - A Comparison between Coupled Cluster Methods and Second-Order Perturbation Theory. *Advances in Quantum Chemistry* **2005**, *48*, 421–433. https://doi.org/10.1016/S0065-3276(05)48022-3.

(6) Sauri, V.; Serrano-Andrés, L.; Shahi, A. R. M.; Gagliardi, L.; Vancoillie, S.; Pierloot, K. Multiconfigurational Second-Order Perturbation Theory Restricted Active Space (RASPT2) Method for Electronic Excited States: A Benchmark Study. *J Chem Theory Comput* **2011**, *7* (1), 153–168. https://doi.org/10.1021/ct100478d.

(7) Andersson, K.; Roos, B. O. Excitation Energies in the Nickel Atom Studied with the Complete Active Space SCF Method and Second-Order Perturbation Theory. **1992**, *191* (6), 507–514. https://doi.org/10.1016/0009-2614(92)85581-t.

(8) Figgen, D.; Peterson, K. A.; Stoll, H. Energy-Consistent Relativistic Pseudopotentials for the 4d Elements: Atomic and Molecular Applications. *Journal of Chemical Physics* **2008**, *128* (3), 034110. https://doi.org/10.1063/1.2822992.
